# Consumer responses and determinants in geographical indications agricultural product consumption: A ten-year systematic review

**DOI:** 10.12688/f1000research.158225.1

**Published:** 2024-11-22

**Authors:** Ailin Tan, Sharizal Bin Hashim, Jiaqi Zuo, Jianyu Cheng

**Affiliations:** 1 Faculty of Economics and Management, Universiti Kebangsaan Malaysia, Bangi, Selangor, 43600, Malaysia; 2 Students affairs office, Chongqing Zhongyi Vocational and Technical School, Wanzhou, Chongqing, 404020, China

**Keywords:** Determinants, Consumption, Geographical Indication, Agricultural Products, Systematic Review.

## Abstract

This article explores the determinants of the consumption of geographical indication agricultural products through a ten-year systematic review. In recent years, the demand for healthier and safer products from consumers has been continuously increasing owing to the influence of the geographical indication of agricultural products. Geographical indication products, a type of label that associates food with specific regions, occupy a certain position in the global food market. This article aims to answer research questions about the determinants of the consumption of geographical indication agricultural products and provide a comparative analysis of five literature reviews related to this topic. We collected and processed relevant research data through a systematic literature review and provided transparent, complete, and accurate explanations using the PRISMA criterion. The research results reveal the consumer response to the geographical indication of agricultural products and the determining factors of different responses. The research in this article is of great significance for understanding the consumption trends and important information of geographical indication of agricultural products, helping enterprises better understand consumer behavior, and improving the income of farmers, producers, and enterprises.

## 1. Introduction

### 1.1 Background

In recent years, the experience of protected origin designation (PDO) foods has influenced the growing demand for healthier and safer products (not only from an environmental point of view) and has given consumers the feeling that they are consuming genuine and high-quality products ( Fotopoulos & Krystallis, 2001). Locally sourced food products are present in the increasingly globalized global food market. Prominent examples of such products include geographical indication (GI) products that associate food with a specific region ( People, 2009). Geographical indications for such labels. Geographical indications (GIs) are geographical indications that indicate the origin of foodstuffs, the characteristics of foodstuffs, and their links with the production area of foodstuffs. The geographical indication is “a symbol” that identifies food as traditional, regional, local, or national due to its unique quality, reputation, or other characteristics ( EU, 2012). As defined by the World Intellectual Property Organization (WIPO, n.d.), a geographical indication (GI) is a symbol used for a product with a specific geographical indication that stands for the quality or reputation of that origin.

PDOs and PGIs can aid in the revitalization of rural areas; to safeguard and promote the diversity of local crops and rural landscapes, enhance social cohesion, it is essential to engage in cereal production and appropriately reward local communities ( Communities, 2008). PDO certification will provide a continuous impetus for entrepreneurship and local development ( Borg & Gratzer, 2013; Vakoufaris, 2010). Considering the conditions and link with food production, GI labels help to further revitalize the rural environment and increase farmers’ incomes, and they can use these labels to profile their professions, thus increasing their added value ( Sanz Cañada & Macías Vázquez, 2005; Tregear, Arfini, Belletti, & Marescotti, 2007).

There is an extensive literature on food in countries of origin e.g. ( Insch, Williams, & Knight, 2016; Newman, Turri, Howlett, & Stokes, 2014; Ozretic-Dosen, Skare, & Krupka, 2007), but there is little research on the determinants of consumption of geographical indication agricultural products as a systematic literature review.

### 1.2 Research question

Given that geographical indications are increasingly recognized by consumers, producers, and businesses worldwide, as well as their fundamental role in adding value to agricultural products, it is crucial to systematically study how geographical indications can be combined with consumer responses in terms of theory and consumption determinants. With the results of this review, interested researchers can gain a better understanding of the trend theory and important information on the consumption of geographically indicated agricultural products, which can help businesses understand consumer behavior at a deeper level, use geographical indications correctly, and improve the income of farmers, producers, and enterprises. A preliminary search of this topic yielded five similar literature reviews. The comparative differences between the five studies are shown in Table 1.

**Table 1.** A Comparative analysis of relevant review.

Reference	Covered years	Research topics	Products origin	Products type	Countries	Limitation
( Thøgersen, 2023)	2010-2023	Consumer product evaluation and choices	Country of origin OR region of origin OR PDO OR PGI	Food, milk, meat, rice, wheat, potatoes, tomatoes, dairy, honey, fruit, vegetables, fish, beef, olive, eggs	OECD countries	No mention of developed and developing countries
( Marion, Luisa, & Sebastian, 2023)	Before 2020 (inclusive)	Adoption GIs by small- and medium-scale enterprises	Geographical IndicationsGIs, origin labels quality food labels	Food crafts	All	Study on small-and medium-scale enterprises not consumers
( Deselnicu, Costanigro, Souza-Monteiro, & McFadden, 2013)	Before 2013	Price premiums in GI products	GIs	Agricultural and food products	All	A meta-analysis
( Glogovețan, Dabija, Fiore, & Pocol, 2022)	2015-2021	Consumer Perception and Understanding	PDO, PGI and TSG labels	Agri-food products	Italy, Poland, Lithuania, Slovakia, Romania, Ukraine, Hungary, Spain, Portugal, Greece, Germany, and South Korea	Omit pertinent material
( Chifor, Arion, Isarie, & Arion, 2022)	Before2022 (inclusive)	CertifiedRomanian products	GIs, PDO and PGI	Agricultural and food products	Romania	Only investigated Romania

As shown in Table 1, current systematic reviews have not provided information on general trends, theoretical positions, determining factors of consumer responses, or consumer processing of geographic indication information in agricultural product consumption. Limited by the countries or regions studied, this has led to certain regional limitations in previous research on the consumption of geographically indicated agricultural products. Therefore, the purpose of this study is to bridge this gap by addressing the following research questions:

RQ1: What were the research trends?

RQ2: What research designs were used?

RQ3: What theories were grounded upon or adopted?

RQ4: What are the responses of consumers to the geographical indications of agricultural products, and what factors determine different responses?

RQ5: What information about the geographical indications of agricultural products affects consumers? Positive or negative impacts or others.

## 2. Search strategy and study selection

### 2.1 Data collection and processing

We adopted a systematic literature review method to explore the literature on the consumption of geographical indications of agricultural products and food. The systematic literature review method has many advantages over traditional methods, Because it can systematically, transparently, and repetitively synthesize literature ( Tranfield, Denyer, & Smart, 2003). Reducing bias and chance effects is a strength of systematic literature reviews, a viewpoint that has been confirmed in previous research, while the legitimacy of data analysis is also enhanced in systematic literature reviews ( Reim, Parida, & Örtqvist, 2015). All the benefits result in research outcomes being enhanced in ways possibly unforeseen and further providing more of the foundation set in order to draw a conclusion ( Reim et al., 2015; Tranfield et al., 2003). It is under guidance by the PRISMA statement how this system review is done, aiming for a transparent as well as comprehensive and precise explanation regarding why such the review took place, which steps happened, and the discoveries made are ( Page et al., 2021). The PRISMA guidelines are primarily designed for the systematic evaluation of studies evaluating the impact of interventions on health ( Page et al., 2021), they must be adapted to the literature being reviewed and the type of review being appropriate for this specific literature institution. The PRISMA method is the best way to prepare a systematic review, as both evaluators and readers can identify the path that the author follows.

The first stage of research strategy is to select ideal keywords to search for relevant research articles, and a preliminary search was conducted using Boolean logic (“Geographical indication” OR “Protected Designation of Origin” OR “Protected Geographical Indication” OR “Geographical indication system” OR “GIs”) AND (“agricultural products” OR “Agri-food” OR “food” OR “agricultural food”) AND (“consumer”).

A search must be conducted in a database that has been proven suitable for a systematic review of academic literature ( Gusenbauer & Haddaway, 2020). The databases “Web of Science” and “Scopus” are used to retrieve publications as they cover a wide range of peer-reviewed scientific articles ( Needham et al., 2020). We also added the conditions with PUBYEAR from 2013 to 2023, and the language was English both in the initial search. To ensure quality and accuracy, only the full texts of the peer-reviewed journal articles were included. The first Web of Science and Scopus search was conducted on 22 December 2023, and 520 publications were identified. The research strings and results are presented in Table 2.

**Table 2.** The search string and the results of article filtering in this study.

Databases	Search string	Number
WoS	(“Geographical indication” OR “Protected Designation of Origin” OR “Protected Geographical Indication” OR “Geographical indication system” OR “GIs”) AND (“agricultural products” OR “Agri-food” OR “food” OR “agricultural food”) AND “consumer”	360
	Document Types: Articles	308
	Language: English	291
	Publish year: 2013-2023	246
Scopus	(“Geographical indication” OR “Protected Designation of Origin” OR “Protected Geographical Indication” OR “Geographical indication system” OR “GIs”) AND (“agricultural products” OR “Agri-food” OR “food” OR “agricultural food”) AND “consumer”	477
	Document Types: Articles	367
	Language: English	343
	Publish year: 2013-2023	274

The second stage included characterizing the inclusion and exclusion criteria to base the final selection of the downloaded articles. The criteria are presented in Table 3.

**Table 3.** Inclusion and exclusion criteria.

Inclusion criteria	Exclusion criteria
Titles and abstracts containing content related to keywords	Titles and abstracts containing content not related to keywords
January 2013 to December 2023	All papers published before January 2013 and after December 2023
Studies should focus on consumer responses to the GI/PGI/PDO Agri-food/products	Studies not focus on consumer responses to the GI/PGI/PDO Agri-food/products
Peer-reviewed journals	Peer-reviewed journals
Written in English	Different from English

**Figure 1.**
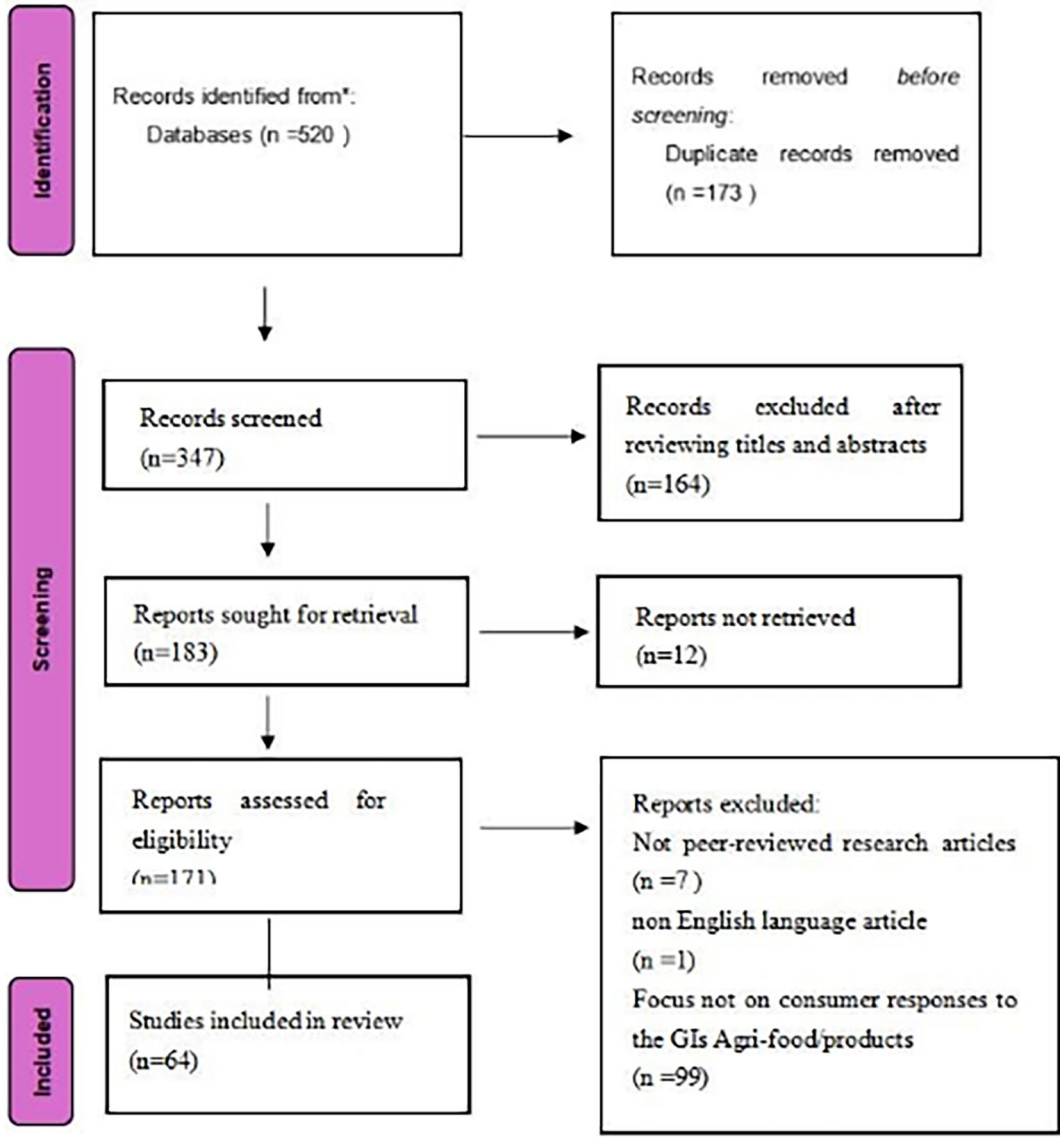
Literature selection process.

Based on this, we conducted the PRISMA review process ( Figure 1), including identification, screening, qualification recognition, and analysis.

A total of 520 studies were imported into Excel Office software. However, after automatically and manually deleting duplicates using this software, we obtained 347 studies that required further review.

After preliminary determination of all titles and abstracts, 164 of 347 articles were excluded because they were not specific to the entirety of agricultural and food products with geographical indications (including wine and spirits) (but on Geographical Indication Policy, Impact on Agriculture, Impact on Supply Chain), reducing the number of relevant publications to 183.

After downloading the full text of the relevant articles, it was found that 12 of them could not be downloaded in full, so there were 171 articles that needed to be read in full.

When conducting full-text reading screening, it was found that seven non-peer-reviewed research articles (books, systematic literature reviews, data analysis, etc.) were excluded. In addition, one non-English-language article was excluded. This number is reduced to 163.

Finally, a screening of the full text of these 163 articles showed that 99 of them were not within the scope of this review (i.e., they lacked raw data from consumer research or did not pay attention to consumer responses to the GI Agri-food/products), resulting in a final sample reduction of 64, labelling ID1-ID64 sequentially (listed in Appendix A).

### 2.2 Data extraction

Reviewed 64 selected studies, at first, the characteristics including titles, authors, year of publication, study design, sample size, citation number, and utilized theoretical framework of the included studies would be summarized. Next, we extracted the determinants of consumption from the included studies. These identified consumptions were further classified based on different variables such as consumer involvement in the response (preferences, cognition, evaluation, purchase intention, etc.). Finally, differences in the extracted data were resolved through discussion.

## 3. Results of review of the studies

### 3.1 Research trends

*3.1.1 Publication timeline and journals*

**Figure 2.**
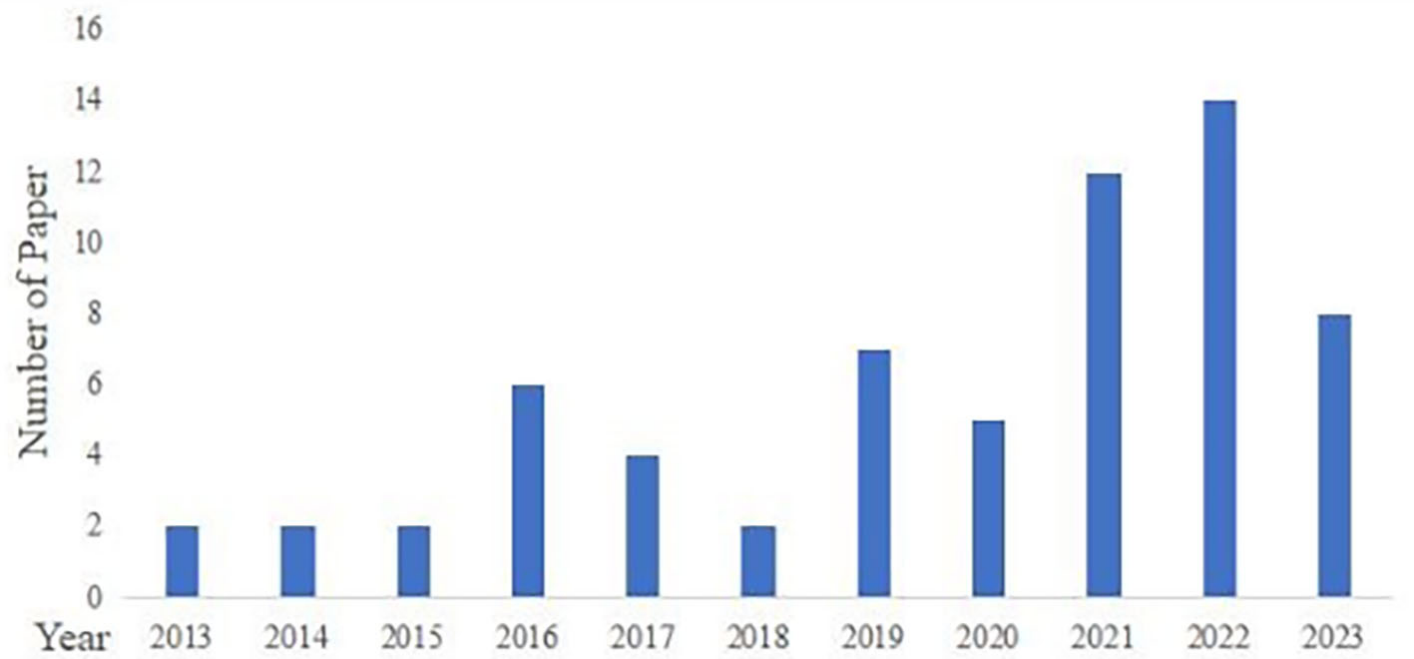
Distribution of publications.

These 64 articles were published between 2013 and 2023 ( Figure 2). From 2013 to 2020, the number of published papers per year remained stable, with no more than seven papers per year. However, starting in 2021, the number of published papers began to increase to 14 by 2022, indicating that an increasing number of scholars have been paying attention to this field.

**Figure 3.**
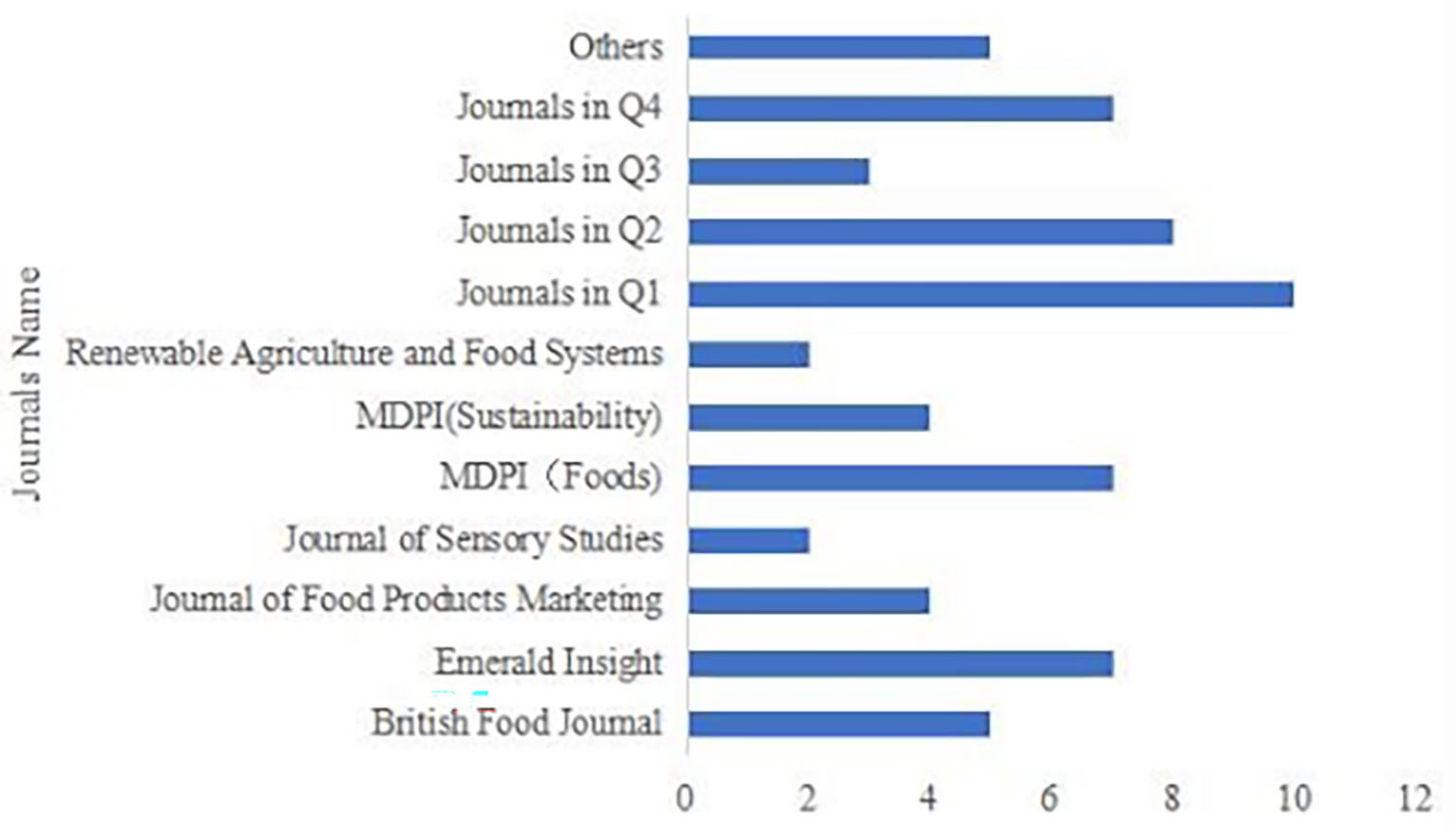
Journals distribution of publications.

These 64 articles were published in 40 different journals (see Figure 3). Among them, 33 journals published only one article each, so they were classified by querying their JIF Quartile (Q1, Q2, Q3, Q4, and others). The journals with the most published papers are MDIP (Foods) and Emerald Insight, each with seven papers. Five articles were from the British Food Journal, four from the Journal of Food Products Marketing, and four from MDIP (Sustainability). In comparison, there are relatively few publications in other journals, with approximately to 2-3 articles.

*3.1.2 Article’s citation number*

Although this list is relatively long, it is clear that there are highly cited and low-cited studies (see Appendix A). Although this list is relatively long, it is clear that there are highly cited and low-cited studies. The article with the highest number of citations is 168 ( Gracia & de-Magistris, 2016), followed by 95 ( Marcoz, Melewar, & Dennis, 2016) and 60 ( Bryla, 2017). They all come from top journals, and the research mostly focuses on consumer attitudes and preferences towards food labelling and certification. All involve the origin of food, the impact of certification and labelling on consumers, and value. However, literature with fewer citations is cited approximately 0 to 10 times ( Çukur, Kizilaslan, Kizilaslan, & Çukur, 2022; Nilgün-Doğan & Adanacıoğlu, 2022; Papoutsi, 2023) etc. Some articles were not published in top-tier journals or had been published for a relatively new period of time.

### 3.2 Research design

*3.2.1 Study setting*

The geographical scope of this study covers many countries and regions, including Italy, Spain, Türkiye, and Serbia, which have the largest number of studies related to geographical indications, as shown in Appendix B. Italy has 26 studies (40%), Spain has six studies (9%), Türkiye has five studies (7%), Serbia has four studies (6%), and other countries have a total of 38% of relevant studies.

A variety of methods were used for data collection, including interviews, questionnaires, internet experiments, and in-depth interviews. Interviews were conducted through a professional networking platform and business emails, and questionnaire surveys and experiments were conducted using software such as statistical analysis tools, data mining, and machine learning. Observational techniques, semi-structured interviews, and household scanning panel data were used.

The analysis methods include various statistical analysis and modeling techniques, such as regression analysis, factor analysis, cluster analysis, structural equation modeling, discrete choice experiments, and multilevel logistic regression. The research tools included qualitative and quantitative survey questionnaires, as well as experimental auctions, selection experiments, and direct ranking preference methods.

*3.2.2 Research methods*

Among the 64 papers, 48 were quantitative, 11 were qualitative, and 5 were mixed. It can be seen that scholars tend to prefer practical quantitative research when studying the relationship between geographical indications and consumers.

*3.2.3 Unit of sample*

From these 64 articles, it can be observed that various sample units were used in different studies. These sample units can be divided into individuals, organizations, and specific target populations.

At the individual level, different individuals were selected as sample units, such as Italian consumers (ID12, ID14, ID16, ID21, ID22, ID26, I27, ID32, ID41, ID43, ID45, ID47, ID54, ID55, ID56, ID59, ID61, ID62, ID64), students from Brazil and Italy (ID2, ID5, ID10, ID18), and Spanish consumers (ID3, ID17, ID19, ID42, ID44, ID50).

At the organizational level, some studies have selected specific organizations as sample units, such as Italian companies (ID1, ID25), Serbia, and producers and distributors of the Mediterranean area (ID20, ID31).

In addition, some studies have conducted sample selection targeting specific populations. For example, research targets consumers with specific attributes or characteristics, such as highly engaged consumers (ID33), Italian Fontina cheese consumers (ID41), Brazilian coffee experts (ID13), and consumers of cheese, ham, and honey in Slovenia (ID19).

### 3.3 Theories

According to Zydev and Warner, theories can be coded into one of three types: grounded theoretical foundations, cited theoretical foundations, or theoretical foundations that are not provided ( Zydney & Warner, 2016).

*3.3.1 Grounded theoretical foundations*

Among the 64 papers, 25 (40%) provided clear statements about the theories used, as detailed in Appendix B. These theories cover multiple fields such as information economics, psychology, and behavioral science. This indicates that research on consumers of GIs agricultural products integrates the latest developments in economics, psychology, and behavioral science research.

*3.3.2 Cited theoretical foundations*

Of the 64 papers, 16 (25%) cited the theoretical analysis results. However, these theories have not been directly applied to the consumption of GIs agricultural products. Among the theories cited, the planned behavior theory, value perception theory, and quality theory are the most commonly cited, indicating that studying the behavior and intention of consuming GIs agricultural products from a consumer perspective has been emphasized in these studies. The second most cited theory is the ethnocentrism theory. Two papers (ID40 and ID50) cite the principles of Ethnic Preference and Geographic Source Identification of this theory, supporting consumer preferences for local products and traditional manufacturing methods and emphasizing the importance of geographic sources as a competitive tool, especially for small producers. Other cited theories include hedonic theory (ID30).

*3.3.3 Theoretical foundations not provided*

22 papers (35%) did not cite any theories to guide their learning or research design.

### 3.4 Consumers responses and the determinants

According to these 64 articles, consumers have different responses to geographical indications of agricultural products. After studying these articles, it was found that willingness to pay (WTP), preference, cognition, and purchase intention had the highest consumption responses. In addition, there were also some responses related to evaluation, consumption, perceived risk, attitude, willingness to pay a premium price (WTPP), etc.

*3.4.1 Willingness to pay and the determinants*

There are 12 articles on willingness to pay, of which 10 have demonstrated that the geographical indication of agricultural products can have a significant positive impact on consumer willingness to pay. When agricultural products are given geographical indications, PDO, or PGI, consumers are more willing to pay for them (ID3, ID5, ID8, ID15, ID18, ID21, ID27, ID34, ID41, ID47, ID50, and ID64).

In these ten articles, the determining factors mainly included sociographic characteristics (3,34, product particle, perception, title, price, color, information, objective and subjective authenticity, imitation, style, and type; additional information; sensory features; and technological use.

In contrast, two articles argue that geographical indications do not have an impact on consumer purchase intention (ID15 and ID47). Papoutsi (2023) believes that consumers are more willing to pay high prices for olive oil with organic certification, and when multiple labels are introduced, they may even show a negative willingness to pay. Stiletto and Trestini (2022) believes that consumers are willing to pay higher prices for products with the “Organic” and “Mountain Product” logos, regardless of whether the product has PDO certification. However, when applying these attributes to PDO products, consumers are less willing to pay, indicating a potential overlap compared to non-PDO products. The determining factors of these two articles depend mainly on whether agricultural products adopt geographical indications.

*3.4.2 Preferences and the determinants*

There were 11 articles on preferences (ID2, ID9, ID12, ID19, ID22, ID24, ID44, ID55, ID57, ID59, and ID63). Artêncio, Giraldi, and de Oliveira (2022) found that women are more sensitive to the origin, while men prefer coffee with geographical indications. Lambarraa-Lehnhardt, Ihle, and Elyoubi (2021a); Rabadán, Martínez-Carrasco, Brugarolas, and Bernabéu (2021); and Skubic, Erjavec, and Klopcic (2018a) indicate that agricultural products with geographical indications are more favored by consumers. Twelve found that consumers attach great importance to navel oranges with information on their freshness, taste, and origin.

The determining factors include sociodemographic characteristics, level of participation, product attribute characteristics, sensor attributes, freshness, taste, place of origin, distance between geographical origin and consumer destination, price, production method, brand/PGI, and spiciness (taste change).

*3.4.3 Purchase intention and the determinants*

There were 11 studies on purchase intention (ID4, ID14, ID17, ID23, ID26, ID33, ID 38, ID45, ID49, ID 51, and ID61). Consumer purchase intention is influenced by various factors, and agricultural products with geographical indications have significant purchase intentions owing to their differences and characteristics. ID14 ID17 consumers’ understanding of geographical indications, origin, and other information can increase their trust in and satisfaction with the product. ID33 products with geographical indication certification are guaranteed to be unique and of high quality through strict quality control, giving consumers greater confidence in them. ID55 The impact of geographical indications and regional origin on consumers is positive as consumers are more inclined to purchase products with geographical indications, especially those from specific regions. ID23 there is a positive correlation between consumers’ attachment to the place and their identification with traditional production methods as well as their willingness and frequency to purchase food products with geographical indications. ID61 consumers’ sense of identification and social identity towards earthquake-stricken areas encourages them to adopt united and mutually supportive behaviors, supporting the recovery of affected areas and strengthening social relationships. ID4 Greek consumers have an insufficient understanding of the labelling of certified products, which has had an impact on their willingness to purchase.

The determining factors include origin, health declaration, labelling, trust, perceived quality, place attachment, perceived behavioral control (PBC), attitude, subjective norms, preferences, perceptions, transparency, cognition, preferences, perceived behavioral control, sense of belonging, and sociodemographic.

*3.4.4 Others and the determinants*

Among the remaining 30 articles (Table 4) (Extended data), there are studies on loyalty (ID1), ethnocentrism (ID32), perception and evaluation (ID40), and word-of-mouth communication (ID58); however, the consumer responses for each of these categories are limited to one study, and the number of studies is relatively small. However, this article on consumer behavior (ID20) does not specify which consumer behaviors it is. In addition, there are approximately to 2-3 related studies on consumption (ID6, ID7), attitude (ID16, ID25, ID28), consumer participation (ID25, ID31), price (ID30, ID43, ID 62) and willingness to pay additional fees (ID29, ID39).

### 3.5 Information and effect

*3.5.1 Related information mentioned*

The 64 documents clearly indicate that information on agricultural products with a geographical indication has an impact on consumers. One of the most frequently mentioned is information about geographically labelled agricultural products themselves. This concerns 19 articles (ID2, ID4, ID5, ID8, ID10, ID17, ID25, ID26, ID27, ID33, ID35, ID36, ID37, ID38, ID42, ID43, ID56, ID57, ID60), including packaging (ID2), product quality (ID26, ID37, ID42, ID57), Internal attributes such as appearance, colour and taste (ID17, ID36, ID37, ID56), and the business culture and production methods of the product are also paid due attention (ID8, ID25, ID27, ID37, ID38, ID43). Interestingly, ID10 mentions advertising and ID11 the storyline. The second most frequently mentioned information was labelling, which was mentioned in 10 articles (ID2, ID3, ID13, ID14, ID15, ID26, ID42, ID44, ID54, and ID55). Next are certifications by official bodies (ID3, ID15, ID33, ID35, ID41, ID47, and ID56). The impact of certification on consumers is mentioned in these seven articles.

*3.5.2 The effect on consumers*

In general, all types of information about the geographical indications of agricultural products have a positive impact on consumers in most cases. More than 31% of the 64 articles analyzed explicitly mentioned this view. Consumers are more willing to pay a premium for geographical indications of agricultural products (PDO) to become the main influence, ID3 explanation is the PDO can better enhance the attractiveness of local characteristics, ID5 consumer perception and attitude towards information about geographical indications, ID14, ID26, ID36, ID43, and ID47 about their product characteristics more transparent and accurate information; ID15, ID, ID33, ID35, ID38, ID44, ID51, ID52, ID53, geographical indications, and country of origin have a positive influence on consumers. Consumers tend to actively treat products based on their geographical indications. However, ID46 believes that for consumers in Slovenia, product quality and its impact on health are more important than origin labels.

Another effect is an increase in consumer perceptions, trust, and satisfaction with the product. ID2 points to the formation of perceptions and expectations of the product, will have a positive effect, ID4 consumers’ understanding of the geographical indication and the origin of products can increase their confidence and satisfaction with the product, can be presented in the form of images or text, to reduce consumers’ perception of risk in food consumption, the ID10 gives consumers a good feeling about the origin and how the product is produced, ID14 appropriate information can help consumers to distinguish and identify products with geographical indication, And to increase the confidence and recognition of these products, ID17 consumers perceive a higher quality of the product’s inherent characteristics, The greater their confidence in the product, The presence of geographical indications ID33 gives consumers greater confidence in the products they buy, they believe that these products have a higher quality than unprotected products, ID38 consumers believe that products with geographical indications have a higher quality and better flavour than other products, the presence of geographical indications gives consumers greater confidence in the reliability and quality of the products.

The effect of the geographical indication of agricultural products on consumers also varies in terms of demographic characteristics. ID2 indicates that women are more sensitive to origin than men. Men prefer coffee with geographical indication; the origin of ID11 coffee has a significant effect on the sensory perception of professional coffee tasters, but no significant effect on the satisfaction rating; ID13 consumers by age, gender, and education level; sensory preference has some significant differences between the analyses.

However, the survey revealed negative effects in the research of some scholars. ID15 consumers are willing to pay higher prices for extra virgin olive oil with organic certification, and the low importance of PDO certification, ID35 Due to the high price of GI rice in the current market, consumers may suffer utility losses with this certification, ID47 consumers are willing to pay higher prices for products with the logo “organic” and “mountain product,” regardless of whether the product has PDO certification. When these attributes are applied to a PDO product, consumers are less willing to pay. The introduction of quality labelling by ID54 can reduce the perceived quality of other products with a geographical indication.

## 4. Discussion

### 4.1 Research trends

This systematic review examined research trends related to the consumption of geographical indication agricultural products, taking into account various factors such as publication timeline, journals, article citation number, research design, study setting, unit of sample, and theories employed. Analysis of these trends provides valuable insights into the current state of research in this field.

Regarding the publication timeline, the review considered studies published within a specific period ranging from the past ten years. This timeframe allowed the inclusion of recent research and ensured the relevance of the findings to the current context. By encompassing studies published over a substantial period, this review captured the evolution of research trends in the field of geographical indication consumption.

The systematic review focused on articles published in reputable and authoritative journals within the fields of agriculture, food studies, and consumer behavior. Journals with a high impact factor and rigorous peer review processes were prioritized to ensure the inclusion of high-quality and influential research.

The article’s number of citations served as an indicator of research significance and influence. The systematic review considered studies with a higher citation count, suggesting that these studies have made notable contributions to the understanding of geographical indication consumption. By including highly cited articles, the review incorporated influential research that has shaped the field.

The research designs employed in the selected studies were also considered. This systematic review considered a range of research designs, including quantitative, qualitative, and mixed-methods approaches. This approach ensured a comprehensive analysis of the various methodologies used in researching geographical indication consumption.

Study settings, including geographical location and national context, were another crucial aspect considered in this review. The inclusion of studies conducted in diverse settings allowed for a broader understanding of geographical indication consumption across different regions and cultural contexts. This consideration enhances the generalizability and applicability of the findings.

The unit of sample, such as individual consumers (ie.ID2,ID3,ID15.etc), households (ID30,ID42,ID59.etc), or businesses (ID31,ID48.etc), were also taken into consideration. By examining studies that focused on different units of the sample, the review provided insights into the various perspectives and levels of analysis in geographical indication consumption research.

Finally, the theories employed in the selected studies are considered to assess the theoretical foundations of the research. This systematic review encompasses studies grounded in various theoretical frameworks, such as consumer behavior theories, economic theories, and sociocultural theories. This comprehensive approach allows for a nuanced understanding of the factors influencing geographical indication consumption.

Overall, the analysis of the publication timeline, journals, citation numbers, research design, study settings, unit of sample, and theories provided a comprehensive overview of the research trends in the field of geographical indication consumption. These considerations ensured the inclusion of influential and diverse studies, thus contributing to a robust understanding of the topic.

### 4.2 Responses and determinants

The systematic review synthesized the existing literature to examine consumer responses to geographical indications of agricultural products and the factors that influence these responses. These findings suggest that geographical indications have a significant impact on consumer behavior and perception (i.e. ID14, ID16, ID17, ID20 etc.). Consumers tend to associate geographical indications with higher quality, authenticity, and cultural significance, leading to increased trust and preference for these products (i.e. ID16, ID21, ID23 etc.). The determinants of consumer responses to geographical indications include factors such as product quality, reputation, familiarity, trust in labelling systems, and personal values (i.e. ID39, ID54, ID61 etc.). Additionally, factors such as price, availability, and marketing strategies also influence consumer decision-making (i.e. ID30, ID43, ID62). Understanding these determinants is crucial for businesses and policymakers to effectively leverage geographical indications and meet consumers’ expectations.

### 4.3 Information and effects

The findings from the analysis of the 64 documents revealed that information related to agricultural products with geographical indications has a significant impact on consumers. The most frequently mentioned information is about the products themselves, including packaging, product quality, and internal attributes such as appearance, color, and taste, as well as the business culture and production methods associated with the product (i.e. ID2, ID 8, ID17, ID25, ID27 etc.). This indicates that consumers are highly influenced by the specific characteristics and attributes of the geographical indications of agricultural products.

Labelling has also been identified as a crucial factor affecting consumer behavior. Consumers pay attention to labels that indicate the geographical origin of the products, as well as certifications provided by official bodies. These labels and certifications contribute to increasing consumer confidence and trust in the products, as well as their willingness to pay a premium for them (i.e. ID3, ID4, ID5, ID10, ID41, ID47 etc.).

Overall, the impact of the geographical indication of agricultural products on consumers is predominantly positive (ID10, ID14). The presence of geographical indications enhances the attractiveness of products, increases consumer perception and trust, and leads to higher satisfaction levels (ID4, ID10, and ID36). Consumers actively seek products with geographical indications and perceive them to have higher quality and better flavor than non-labelled products. Information provided through geographical indications helps consumers make informed choices and differentiate these products from others in the market.

The effect of geographical indications on consumers may vary depending on their demographic characteristics. For example, women were found to be more sensitive to the origin of products than men (ID2). Furthermore, age, sex, and education level can lead to differences in sensory preferences among consumers (ID7 and ID13).

However, it is important to note that some scholars have identified certain negative effects associated with the geographical indication of agricultural products (ID15 and ID47). These include instances in which consumers may be unwilling to pay higher prices for products with specific certifications or labels, such as PDO certification. Additionally, the introduction of quality labelling for certain products may inadvertently reduce the perceived quality of other products with geographical indications.

## 5. Conclusion

In conclusion, this systematic review sheds light on the determinants of the consumption of geographically indicated agricultural products and the corresponding consumer responses. These findings highlight the growing interest in geographical indications and their potential to add value to agricultural products. However, the review also revealed research gaps, such as the limited focus on the determinants of consumption and regional limitations in previous studies. Future research should address these gaps through comprehensive cross-national studies that explore consumer behavior, preferences, and decision-making processes related to geographical indications. In addition, it is crucial to ensure accurate and transparent information about geographical indications to build consumer trust and avoid confusion. Overall, understanding consumer responses and improving information dissemination can contribute to the successful implementation and utilization of geographical indications in the agricultural food market.

Ethical & consent: Ethical approval and consent were not required.

## Data Availability

No data are associated with this article Figshare: Flow of PRISMA.jpg, https://doi.org/10.6084/m9.figshare.27627237.v1 ( Tan, 2024b). Data are available under the terms of the CC0 1.0 Universal. Figshare: Dataset.PRISMA checklist, https://doi.org/10.6084/m9.figshare.27627324.v1 ( Tan, 2024a). Data are available under the terms of the CC0 1.0 Universal. Figshare: Consumers responses and determinants, Details of the reviewed studies, List of theoretical foundations and Methodologies cited in reviewed studies, https://doi.org/10.6084/m9.figshare.27627480.v1 ( Tan, 2024c). The project contains following data:
1.
Table 4, 2. Appendix A, 3. Apeendix B Data are available under the terms of the CC0 1.0 Universal.

## Appendix A. Details of the reviewed studies.

**Table:**

ID	Title	Journal of Publication	Publication Year	Total Citations
ID1	Marketing geographical indication products in the digital age: a holistic perspective ( Bartoli, Bonetti, & Mattiacci, 2022)	British Food Journal	2022	4
ID2	A cup of black coffee with GI, please! Evidence of geographical indication influence on a coffee tasting experiment ( Artêncio et al., 2022)	Physiology & Behavior	2022	5
ID3	Labels for a Local Food Speciality Product: The Case of Saffron ( Sanjuan-Lopez & Resano-Ezcaray, 2020)	Journal of Agricultural Economics	2020	24
ID4	Consumers’ intention to buy protected designation of origin and protected geographical indication foodstuffs: the case of Greece ( Likoudis, Sdrali, Costarelli, & Apostolopoulos, 2016)	International Journal of Consumer Studies	2016	27
ID5	Brand Coopetition with Geographical Indications: Which Information Does Lead to Brand Differentiation? ( Dentoni, Tonsor, Calantone, & Peterson, 2013)	New Medit	2013	16
ID6	AN ANALYSIS OF THE FACTORS AFFECTING THE CONSUMPTION OF GEOGRAPHICALLY INDICATED PRODUCTS USING DECISION TREE AND ARTIFICIAL NEURAL NETWORKS ( Çukur et al., 2022)	Journal of Animal & Plant Sciences	2022	N/A
ID7	CONSUMERS PERCEPTION AND BEHAVIOUR TOWARDS GEOGRAPHICAL INDICATION PRODUCTS: THE CASE OF TRADITIONAL PESTIL FROM GUMUSHANE, TURKEY ( Nilgün-Doğan & Adanacıoğlu, 2022)	Agrociencia	2022	N/A
ID8	Consumers’ Preferences for the Consumption of the Fresh Black Slavonian Pig’s Meat ( Jelić Milković et al., 2023)	MDPI (Foods)	2023	4
ID9	How Successful Is Origin Labeling in a Developing Country Context? Moroccan Consumers’ Preferences toward Local Products ( Lambarraa-Lehnhardt, Ihle, & Elyoubi, 2021a)	MDPI (Sustainability)	2021	21
ID10	Product versus region of origin: which wins in consumer persuasion? ( Luceri, Latusi, & Zerbini, 2016)	Emerald Insight	2016	51
ID11	The impact of coffee origin information on sensory and hedonic judgment of fine Amazonian robusta coffee ( Artencio et al., 2023)	Journal of Sensory Studies	2022	2
ID12	Study of Consumer Preferences in Regard to the Blonde Orange Cv. Washington Navel “Arancia Di Ribera PDO” ( Ingrassia, Sgroi, Tudisca, & Chironi, 2017)	Journal of Food Products Marketing	2016	26
ID13	Consumers’ hedonic liking of different labeled and conventional food products in Slovenia ( Skubic, Erjavec, Ule, & Klopcic, 2018b)	Journal of Sensory Studies	2018	17
ID14	Familiar worldwide: how PDO products reflect quality in consumers’ appraisal and behaviour ( Toma, Manta, Morrone, & Campobasso, 2023)	Emerald Insight	2022	6
ID15	Consumer Valuation of European Certification Labels on Extra Virgin Olive Oil: Assessing the Impact of Multiple Labels and Consumer Heterogeneity ( Papoutsi, 2023)	Journal of Food Products Marketing	2023	N/A
ID16	The Value of Region of Origin, Producer and Protected Designation of Origin Label for Visitors and Locals: The Case of Fontina Cheese in Italy ( Marcoz et al., 2016)	International Journal of Tourism Research	2016	95
ID17	Exploring the mediating role of trust in food products with Protected Designation of Origin. The case of “Jamón de Teruel” ( Fandos-Herrera, 2016)	Spanish Journal of Agricultural Research	2016	15
ID18	The effect of information and co-branding strategies on consumers willingness to pay (WTP) for Protected Designation of Origin (PDO) products: the case of pre-sliced Parma Ham ( Arfini & Mancini, 2015)	Progress in Nutrition	2015	11
ID19	Perceptions of geographical indication labels as quality indicators inside and outside the labels’ area of influence: the case of spring fruits ( Rabadán et al., 2021)	Renewable Agriculture and Food Systems	2021	10
ID20	Market-Oriented Sustainability of Sjenica Sheep Cheese ( Filipovic, 2019)	MDPI (Sustainability)	2019	4
ID21	Development and Validation of the Perceived Authenticity Scale for Cheese Specialties with Protected Designation of Origin ( Sidali, Capitello, & Manurung, 2021)	MDPI (Foods)	2021	9
ID22	Understanding the Role of Purchasing Predictors in the Consumer’s Preferences for PDO Labelled Honey ( Di Vita, Pippinato, et al., 2021a)	Journal of Food Products Marketing	2021	19
ID23	The effect of place attachment of geographical indication agricultural products on repurchase intention ( Zhe, Jie, & Yuan, 2023)	Journal of Retailing and Consumer Services	2023	7
ID24	Consumer preferences regarding national and EU quality labels for cheese, ham and honey The case of Slovenia ( Skubic, Erjavec, & Klopcic, 2018a)	Emerald Insight	2017	53
ID25	Communication patterns to address the consumption of PDO products ( Bonetti, Mattiacci, & Simoni, 2019)	Emerald Insight	2019	6
ID26	Choice Drivers for Quality-Labelled Food: A Cross-Cultural Comparison on PDO Cheese ( Menozzi, Giraud, Saïdi, & Yeh, 2021)	MDPI (Foods)	2021	2
ID27	Do consumers really recognise a distinct quality hierarchy amongst PDO sparkling wines? The answer from experimental auctions ( Galletto et al., 2021)	Emerald Insight	2020	11
ID28	CONSUMER ATTITUDES AND HABITS ABOUT PRODUCTS WITH GEOGRAPHICAL INDICATION IN SERBIA ( Ciric, Ciric, Pivac, & Besermenji, 2023)	Економика пољопривредеЕкономика пољопривреде	2023	N/A
ID29	Do South African Consumers have an Appetite for an Originbased Certification System for Meat Products? A Synthesis of Studies on Perceptions, Preferences and Experiments ( Kirsten et al., 2017)	International Journal on Food System Dynamics	2017	15
ID30	Valuing country of origin and organic claim A hedonic analysis of cheese purchases of German households ( Schröck, 2014)	Emerald Insight	2013	48
ID31	Establishing Communities of Value for Sustainable Localized Food Products: The Case of Mediterranean Olive Oil ( Radić, Monaco, Cerdan, & Peri, 2023)	MDPI (Sustainability)	2023	1
ID32	Where was my cup of honey made? PDO honey and sub-regional ethnocentric consumer segments ( Trentinaglia, Cavicchioli, Pocol, & Baldi, 2023)	Emerald Insight	2022	1
ID33	Drivers of high-involvement consumers’ intention to buy PDOwines: Valpolicella PDO case study ( Capitello, Agnoli, & Begalli, 2016)	Journal of the Science of Food and Agriculture	2015	12
ID34	Consumers’ willingness to pay for geographical origin labels: evidence from the Turkish table olive ( Ozkan & Gurbuz, 2023)	researchgate	2022	N/A
ID35	Consumers’ Valuation of Geographical Indication-Labeled Food: The Case of Hom Mali Rice in Bangkok ( Lee, Pavasopon, Napasintuwong, & Nayga, 2020)	Asian Economic Journal	2020	9
ID36	Consumers’ Preference for the Consumption of the Fresh Black Slavonian Pig’s Meat ( Milkovic et al., 2023)	MDPI (Foods)	2023	4
ID37	The Role of GI Products or Local Products in the Environment—Consumer Awareness and Preferences in Albania, Bulgaria and Poland ( Muça, Pomianek, & Peneva, 2022)	MDPI (Sustainability)	2021	21
ID38	Do consumers intend to purchase the food with Geographical Indication? ( Aytop & Cankaya, 2022)	Mediterranean journal of economics, agriculture and environment	2022	2
ID39	MARKET PERSPECTIVES FOR SERBIAN PDO PRODUCTS IN THE REPUBLIC OF SERBIA ( Panin, 2022)	THE CENTRAL EUROPEAN JOURNAL OF REGIONAL DEVELOPMENT AND TOURISM	2022	N/A
ID40	THE EFFECT OF A COUNTRY NAME ON CONSUMERS’ PERCEPTION AND ASSESSMENT OF AGRICULTURAL PRODUCTS WITH PROTECTED DESIGNATION OF ORIGIN ( Rakic, Bugarcic, & Draskovic, 2022)	Economics of Agriculture	2022	1
ID41	Willingness to pay for P.D.O. certification: an empirical investigation ( Garavaglia & Marcoz, 2014)	Int. J. Food System Dynamics	2014	1
ID42	Not Seeing the Forest for the Trees: The Impact of Multiple Labelling on Consumer Choices for Olive Oil ( Pérez, Gracia, & Barreiro-Hurlé, 2020)	MDPI (Foods)	2020	20
ID43	Consumer Preferences for Cheese Products with Quality Labels: The Case of Parmigiano Reggiano and Comte ( Menozzi, Yeh, Cozzi, & Arfini, 2022)	MDPI (Animals)	2022	6
ID44	Consumer preferences for food labeling: What ranks first ( Gracia & de-Magistris, 2016)	Food Control	2016	168
ID45	An investigation into Italian consumers’ awareness, perception, knowledge of European Union quality certifications, and consumption of agri-food products carrying those certifications ( Sampalean, Rama, & Visentin, 2021)	Bio-based and Applied Economics Journal	2021	6
ID46	Consumer awareness of PDO-labelled food in Slovenia ( Skubic, Erjavec, & Klopcic, 2019)	Italian Journal of Animal Science	2019	18
ID47	When less isn’t more and more isn’t less: is there an overlap between “protected designation of origin”, “mountain product” and “organic” in Italy? ( Stiletto & Trestini, 2022)	British Food Journal	2023	4
ID48	Consumers’ awareness of the EU’s protected designations of origin logo ( Goudis & Skuras, 2021)	British Food Journal	2021	29
ID49	Purchasing local and non-local products labeled with geographical indications (GIs) ( Albayram, Mattas, & Tsakiridou, 2014)	Operational Research	2014	20
ID50	Geographical indication food products and ethnocentric tendencies: The importance of proximity, tradition, and ethnicity ( Fernández-Ferrín et al., 2019)	Journal of Cleaner Production	2019	57
ID51	Foreign Geographical Indications, Consumer Preferences, and the Domestic Market for Cheese ( Slade, Michler, & Josephson, 2019)	Applied Economic Perspectives and Policy	2019	28
ID52	Geographical indications for supporting rural development in the context of the Green Morocco Plan: Oasis dates ( Lambarraa-Lehnhardt, Ihle, & Mhaouch, 2021b)	Agricultural Economics/Zemědělská Ekonomika	2021	7
ID53	The perception of EU quality signs for origin and organic food products among Polish consumer ( Bryla, 2017)	Quality Assurance and Safety of Crops & Foods	2017	60
ID54	Vertical differentiation via multi-tier geographical indications and the consumer perception of quality: The case of Chianti wines ( Costanigro, Scozzafava, & Casini, 2019)	Food Policy	2019	24
ID55	EU quality labels in the Italian olive oil market: How much overlap is there between geographical indication and organic production? ( Roselli, Giannoccaro, Carlucci, & De Gennaro, 2018)	Journal of Food Products Marketing	2018	29
ID56	Italian consumers’ awareness, preferences and attitudes about Sicilian blood oranges (Arancia Rossa di Sicilia PGI) ( Selvaggi, Zarbà, Pappalardo, Pecorino, & Chinnici, 2023)	Journal of Agriculture and Food Research	2023	6
ID57	Food traditions and consumer preferences for cured meats: Role of information in geographical indications ( Sgroi, 2021)	International Journal of Gastronomy and Food Science	2021	17
ID58	Geographical indication labelling of food and behavioural intentions ( Chen, 2021)	British Food Journal	2021	7
ID59	Oh my darling clementine: heterogeneous preferences for sustainable citrus fruits ( Di Vita, Vecchio, et al., 2021b)	Renewable Agriculture and Food Systems	2021	12
ID60	Effect of Geographical Indication Information on Consumer Acceptability of Cooked Aromatic Rice ( Arroyo, Hogan, Wisdom, Moldenhauer, & Seo, 2020)	MDPI (Foods)	2020	12
ID61	May trust and solidarity defy food scares? The case of Parmigiano-Reggiano PDO sales in the aftermath of natural disaster ( Menozzi & Finardi, 2019)	British Food Journal	2019	13
ID62	How Much Do Consumers Value Protected Designation of Origin Certifications? Estimates of willingness to Pay for PDO Dry-Cured Ham in Italy ( Garavaglia & Mariani, 2017)	Agribusiness	2017	54
ID63	Understanding Consumers’ Preferences for Protected Geographical Indications: A Choice Experiment with Hungarian Sausage Consumers ( Török, Gorton, Yeh, Czine, & Balogh, 2022)	MDPI (Foods)	2022	18
ID64	Consumer awareness of sustainable supply chains: A choice experiment on Parma ham PDO ( Mazzocchi, Orsi, Zilia, Costantini, & Bacenetti, 2022)	Science of The Total Environment	2022	11
