# Consumer responses and determinants in geographical indications agricultural product consumption: A ten-year systematic review

**DOI:** 10.12688/f1000research.158225.2

**Published:** 2026-02-03

**Authors:** Ailin Tan, Sharizal Bin Hashim, Jiaqi Zuo, Jianyu Cheng

**Affiliations:** 1 Faculty of Economics and Management, Universiti Kebangsaan Malaysia, Bangi, Selangor, 43600, Malaysia; 2 Students affairs office, Chongqing Zhongyi Vocational and Technical School, Wanzhou, Chongqing, 404020, China

**Keywords:** Determinants, Consumption, Geographical Indication, Agricultural Products, Systematic Review.

## Abstract

This article analyzes the determinants of consumers’ purchase and consumption of geographical indication (GI) agricultural products through a ten-year systematic review. There exist growing concerns about food safety, quality, and health, increasing consumer attention to origin labelled foods. GI agricultural products have gained a stable position in global food markets. This study pursues two objectives. First, it identifies and classifies the key determinants of GI agricultural product consumption reported in the literature. Second, it compares insights from five existing review studies on closely related themes to clarify what is already established and what remains contested. Following the PRISMA guidelines, we conducted a transparent and reproducible systematic literature review, including structured searching, screening, and data extraction procedures. The results summarize how consumers respond to GI cues and identify factors associated with heterogeneous responses across contexts. By integrating fragmented evidence, this review clarifies consumption relevant mechanisms and offers implications for GI governance and marketing, helping firms better understand consumer decision making and supporting value capture for farmers, producers, and regional stakeholders.

## 1. Introduction

### 1.1 Background

In recent years, experiences with protected designation of origin (PDO) foods have contributed to a growing demand for healthier and safer products, not only from an environmental perspective. They also give consumers the feeling that they are consuming genuine and high-quality products ( Fotopoulos & Krystallis, 2001).

At the same time, locally sourced food products have become more visible in an increasingly global food market. A prominent example is geographical indication (GI) products, which associate food with a specific region ( EU, 2012). Similarly, the World Intellectual Property Organization ( WIPO, n.d.) defines a GI as a sign used on products with a specific geographical origin, where a given quality, reputation, or other characteristic is essentially attributable to that origin.

PDOs and PGIs can support rural revitalization. They help safeguard and promote the diversity of local crops and rural landscapes and enhance social cohesion, especially when local communities are appropriately engaged and rewarded ( Communities, 2008). PDO certification can also provide continuous impetus for entrepreneurship and local development ( Borg & Gratzer, 2013; Vakoufaris, 2010). More broadly, by strengthening the link between products and their places of production, GI labels can contribute to rural development and increase farmers’ incomes. They also help farmers and producers position their products and increase added value ( Sanz Cañada & Macías Vázquez, 2005; Tregear, Arfini, Belletti, & Marescotti, 2007).

There is extensive literature on country of origin effects for food products ( Insch, Williams, & Knight, 2016; Newman, Turri, Howlett, & Stokes, 2014; Ozretic-Dosen, Skare, & Krupka, 2007). However, research that systematically reviews the determinants of consumption of geographical indication agricultural products remains limited.

### 1.2 Research question

Given that geographical indications are increasingly recognized by consumers, producers, and businesses worldwide, as well as their fundamental role in adding value to agricultural products, it is crucial to systematically study how geographical indications can be combined with consumer responses in terms of theory and consumption determinants. With the results of this review, interested researchers can gain a better understanding of the trend theory and important information on the consumption of geographically indicated agricultural products, which can help businesses understand consumer behavior at a deeper level, use geographical indications correctly, and improve the income of farmers, producers, and enterprises. A preliminary search of this topic yielded five similar literature reviews. The comparative differences between the five studies are shown in Table 1.

**Table 1.** A Comparative analysis of relevant review.

Reference	Covered years	Research topics	Products origin	Products type	Countries	Limitation
( Thøgersen, 2023)	2010-2023	Consumer product evaluation and choices	Country of origin OR region of origin OR PDO OR PGI	Food, milk, meat, rice, wheat, potatoes, tomatoes, dairy, honey, fruit, vegetables, fish, beef, olive, eggs	OECD countries	No mention of developed and developing countries
( Marion, Luisa, & Sebastian, 2023)	Before 2020 (inclusive)	Adoption GIs by small- and medium-scale enterprises	Geographical IndicationsGIs, origin labels quality food labels	Food crafts	All	Study on small-and medium-scale enterprises not consumers
( Deselnicu, Costanigro, Souza-Monteiro, & McFadden, 2013)	Before 2013	Price premiums in GI products	GIs	Agricultural and food products	All	A meta-analysis
( Glogovețan, Dabija, Fiore, & Pocol, 2022)	2015-2021	Consumer Perception and Understanding	PDO, PGI and TSG labels	Agri-food products	Italy, Poland, Lithuania, Slovakia, Romania, Ukraine, Hungary, Spain, Portugal, Greece, Germany, and South Korea	Omit pertinent material
( Chifor, Arion, Isarie, & Arion, 2022)	Before2022 (inclusive)	CertifiedRomanian products	GIs, PDO and PGI	Agricultural and food products	Romania	Only investigated Romania

As shown in Table 1, existing reviews address origin-based labels and geographical indication schemes from complementary but only partially overlapping perspectives, which makes their findings difficult to integrate without a clearer comparative lens. In terms of analytical focus, Thøgersen (2023) is the most consumer oriented and decision focused, synthesizing how origin cues shape consumer evaluation and choice across many food categories. By comparison, Marion, Luisa, and Sebastian (2023) shifts the unit of analysis from consumers to small and medium scale enterprises, emphasizing adoption and implementation of GI and related origin and quality labels, which speaks more to supply side capability than to demand side determinants. Deselnicu et al. (2013) narrows the lens further by treating consumer response primarily as willingness to pay, quantifying GI related price premiums through meta-analysis. This approach is powerful for summarizing average monetary effects, but it abstracts from psychological and contextual mechanisms that explain variation in consumer behavior. Glogovețan et al. (2022) sits between these streams by focusing on consumer perception and understanding of PDO, PGI, and TSG labels, offering insight into the cognitive channel that may underlie purchasing decisions, yet its evidence base is limited to 2015 to 2021 and is reported to omit relevant materials, which weakens completeness. Chifor et al. (2022) provides the most context specific synthesis by focusing on Romania, enabling richer institutional and product level interpretation, but at the cost of cross-country comparability.

Across these reviews, the main trade off is clear. Broad and multi product syntheses, such as Thøgersen (2023), provide coverage but remain concentrated in OECD settings and do not clearly separate developed from developing contexts. Highly specialized reviews, such as Deselnicu et al. (2013), provide strong quantitative aggregation but focus on one outcome dimension and therefore offer limited guidance on non-price determinants. Context rich reviews, such as Chifor et al. (2022), improve depth but limit transferability. Finally, perception focused reviews, such as Glogovețan et al. (2022), clarify label understanding but do not fully connect that understanding to consistent determinants of actual consumption across settings. Overall, prior reviews are fragmented by actor level, outcome type, and geographic scope. What is still missing is a unified, consumer centered synthesis that jointly considers multiple determinants beyond price, evaluates how these determinants vary across contexts, and consolidates the evidence base over a longer time horizon for GI agricultural products.

Therefore, the purpose of this study is to bridge this gap by addressing the following research questions:

RQ1: What are the main research trends in studies on consumers and geographical indication agricultural products during the review period?

RQ2: What research designs and methods have been used in this literature?

RQ3: What theories or theoretical frameworks have been used to explain consumer responses to geographical indications?

RQ4: How do consumers respond to geographical indications on agricultural products, and what factors explain differences in these responses?

RQ5: What types of geographical indication related information influence consumers, and do these influences tend to be positive, negative, or mixed?

## 2. Search strategy and study selection

### 2.1 Data collection and processing

We adopted a systematic literature review to examine research on the consumption of geographical indications (GIs) for agricultural and food products. Compared with traditional narrative reviews, a systematic review enables a structured, transparent, and replicable synthesis of prior studies ( Tranfield, Denyer, & Smart, 2003). It also helps reduce bias and chance effects and strengthens the credibility of the evidence base and the legitimacy of the analysis ( Reim, Parida, & Örtqvist, 2015). As a result, systematic reviews can generate more reliable conclusions and clarify what is known and what remains uncertain in the literature ( Reim, Parida, & Örtqvist, 2015).

This review followed the PRISMA statement to document the rationale, procedures, and key outputs in a clear and traceable manner ( Reim et al., 2015; Tranfield et al., 2003). Although PRISMA was originally developed for systematic reviews of health interventions, its core principles can be adapted to other fields by aligning the reporting items with the objectives and scope of the review ( Page et al., 2021). Using PRISMA therefore improves transparency for both reviewers and readers by making the search and selection process explicit.

The first stage of the research strategy was to identify appropriate keywords and develop search strings. We conducted a preliminary search using Boolean operators: (“Geographical indication” OR “Protected Designation of Origin” OR “Protected Geographical Indication” OR “Geographical indication system” OR “GIs”) AND (“agricultural products” OR “agri-food” OR “food” OR “agricultural food”) AND (“consumer”).

Following recommendations on database selection for systematic reviews ( Gusenbauer & Haddaway, 2020), we searched Web of Science and Scopus because they provide broad coverage of peer reviewed journal publications ( Needham et al., 2020). In the initial search, we limited the publication years to 2013–2023 and restricted the language to English. To ensure quality and comparability, we included only full text peer reviewed journal articles. The Web of Science and Scopus searches were conducted on 22 December 2023, yielding 520 records. The search strings and results are reported in Table 2.

**Table 2.** The search string and the results of article filtering.

Databases	Search string	Number
WoS	(“Geographical indication” OR “Protected Designation of Origin” OR “Protected Geographical Indication” OR “Geographical indication system” OR “GIs”) AND (“agricultural products” OR “Agri-food” OR “food” OR “agricultural food”) AND “consumer”	360
	Document Types: Articles	308
	Language: English	291
	Publish year: 2013-2023	246
Scopus	(“Geographical indication” OR “Protected Designation of Origin” OR “Protected Geographical Indication” OR “Geographical indication system” OR “GIs”) AND (“agricultural products” OR “Agri-food” OR “food” OR “agricultural food”) AND “consumer”	477
	Document Types: Articles	367
	Language: English	343
	Publish year: 2013-2023	274

The second stage included characterizing the inclusion and exclusion criteria to base the final selection of the downloaded articles. The criteria are presented in Table 3.

**Table 3.** Inclusion and exclusion criteria.

Inclusion criteria	Exclusion criteria
Titles and abstracts containing content related to keywords	Titles and abstracts containing content not related to keywords
January 2013 to December 2023	All papers published before January 2013 and after December 2023
Studies should focus on consumer responses to the GI/PGI/PDO Agri-food/products	Studies not focus on consumer responses to the GI/PGI/PDO Agri-food/products
Peer-reviewed journals	Peer-reviewed journals
Written in English	Different from English

**Figure 1.**
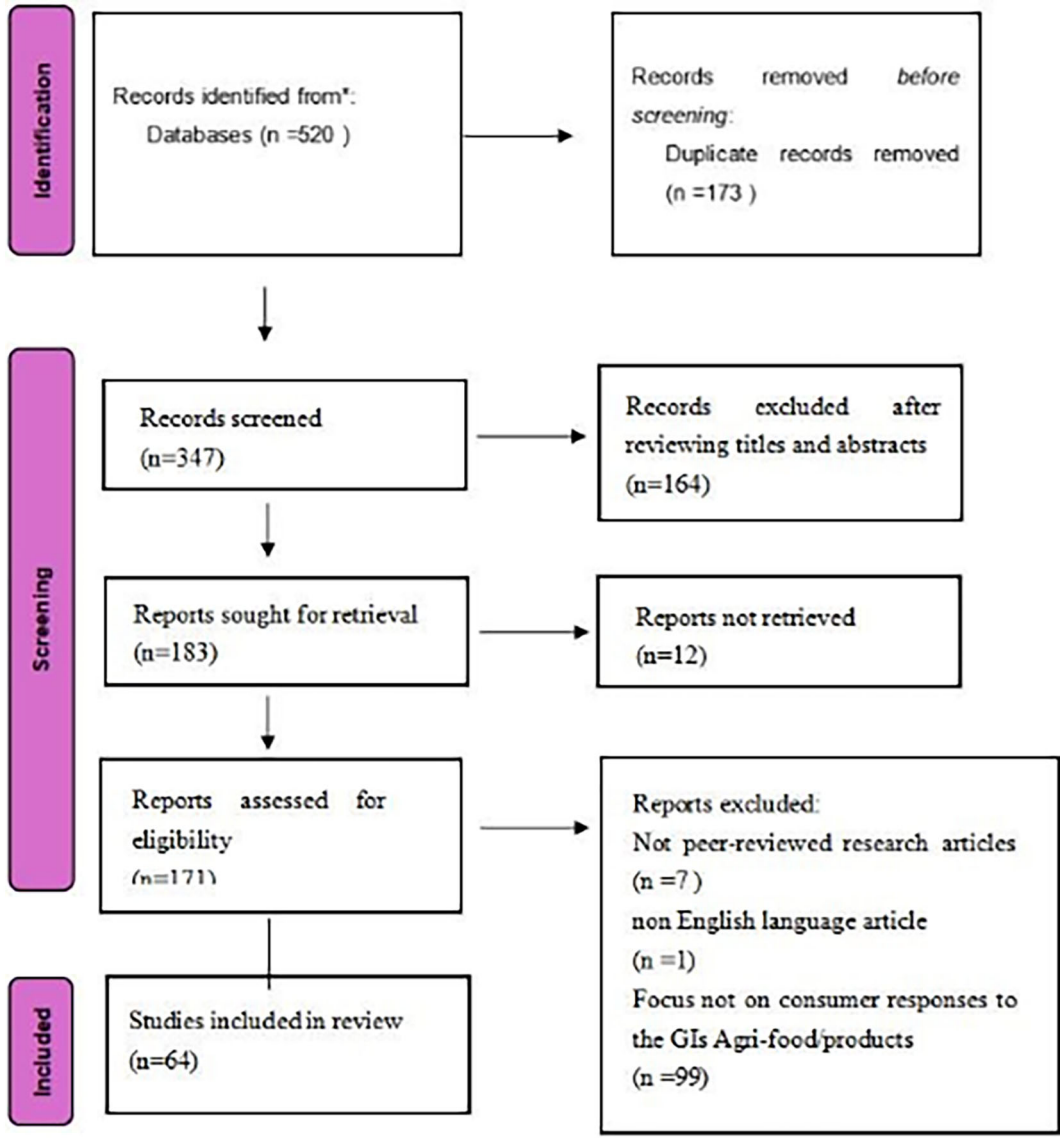
Literature selection process.

Based on this, we conducted the PRISMA review process ( Figure 1), including identification, screening, qualification recognition, and analysis.

The total of 520 records were imported into Microsoft Excel for management and screening. After automated and manual de duplication, 347 unique records remained.

Title and abstract screening were then conducted using predefined eligibility criteria. Studies were retained if they addressed geographical indications and related schemes such as GI, PDO, or PGI in the context of agricultural and food products, including wine and spirits, and if they were relevant to consumer related outcomes. In contrast, 164 records were excluded because they were outside the scope of this review. These excluded records primarily focused on topics such as GI policy and regulation, impacts on agriculture at the sector level, supply chain and logistics issues, or other themes not centered on GI agri food products and consumer responses. After this step, 183 articles were retained for full text assessment.

Full texts were sought for the 183 articles. Twelve articles could not be retrieved in full and were excluded for this reason, leaving 171 articles for full text screening. During full text screening, seven items were excluded because they were not peer reviewed journal articles, including books and review type publications. One article was excluded because it was not published in English. This resulted in 163 articles eligible for the final eligibility assessment.

In the final eligibility assessment, the full texts of the 163 articles were evaluated against the core inclusion criteria of this review, namely that the study reported empirical consumer research and examined consumer responses to GI agri food products. Empirical consumer research was defined as studies providing original consumer level evidence, such as surveys, experiments, choice modelling, or interviews, and consumer responses included outcomes such as perceptions, preferences, willingness to pay, purchase intentions, or choice behaviour. Based on these criteria, 99 articles were excluded because they did not report consumer data or did not analyse consumer responses to GI agri food products. The final sample therefore consisted of 64 studies, labelled sequentially as ID1 to ID64 (listed in Appendix A).

### 2.2 Data extraction

Reviewed 64 selected studies, at first, the characteristics including titles, authors, year of publication, study design, sample size, citation number, and utilized theoretical framework of the included studies would be summarized. Next, we extracted the determinants of consumption from the included studies. These identified consumptions were further classified based on different variables such as consumer involvement in the response (preferences, cognition, evaluation, purchase intention, etc.). Finally, differences in the extracted data were resolved through discussion.

## 3. Results of review of the studies

### 3.1 Research trends

*3.1.1 Publication timeline and journals*

**Figure 2.**
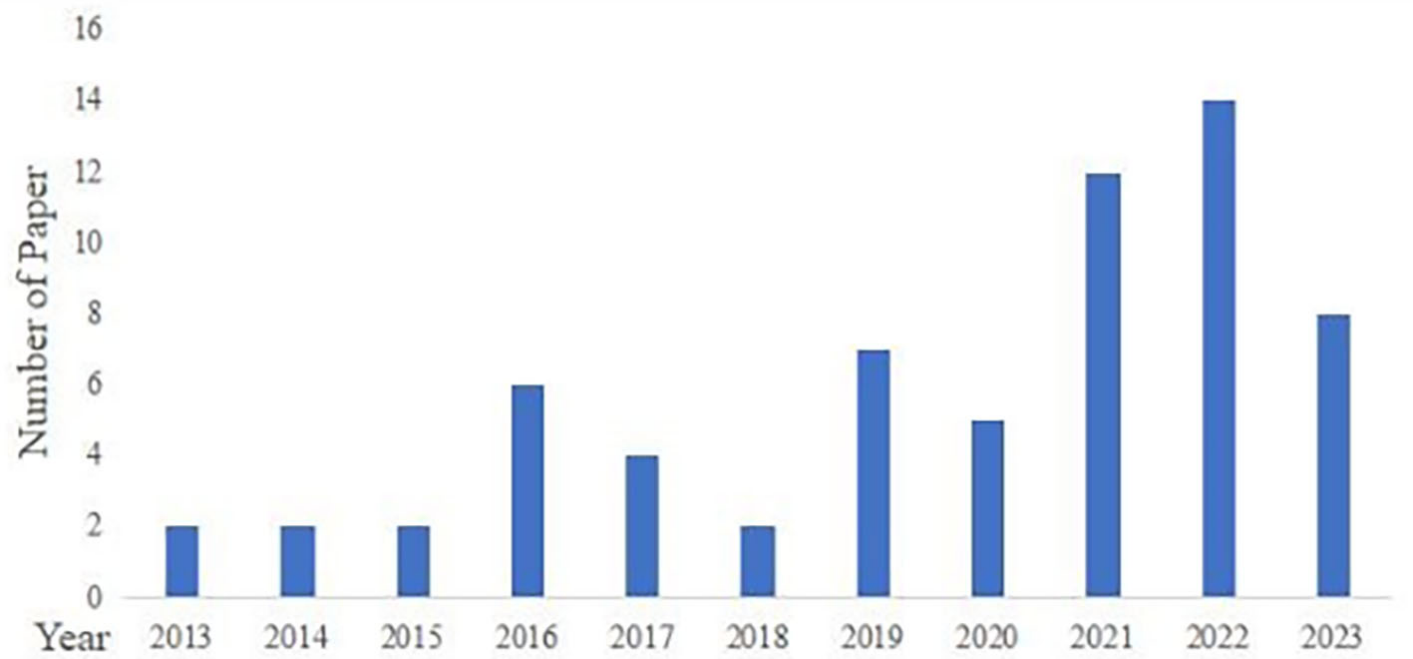
Distribution of publications.

These 64 articles were published between 2013 and 2023 ( Figure 2). From 2013 to 2020, annual publication output remained relatively stable, with no more than seven papers per year. From 2021 onward, the number of publications increased markedly, reaching 14 papers in 2022. This pattern suggests growing scholarly attention to consumer related research on GI agri food products in recent years.

**Figure 3.**
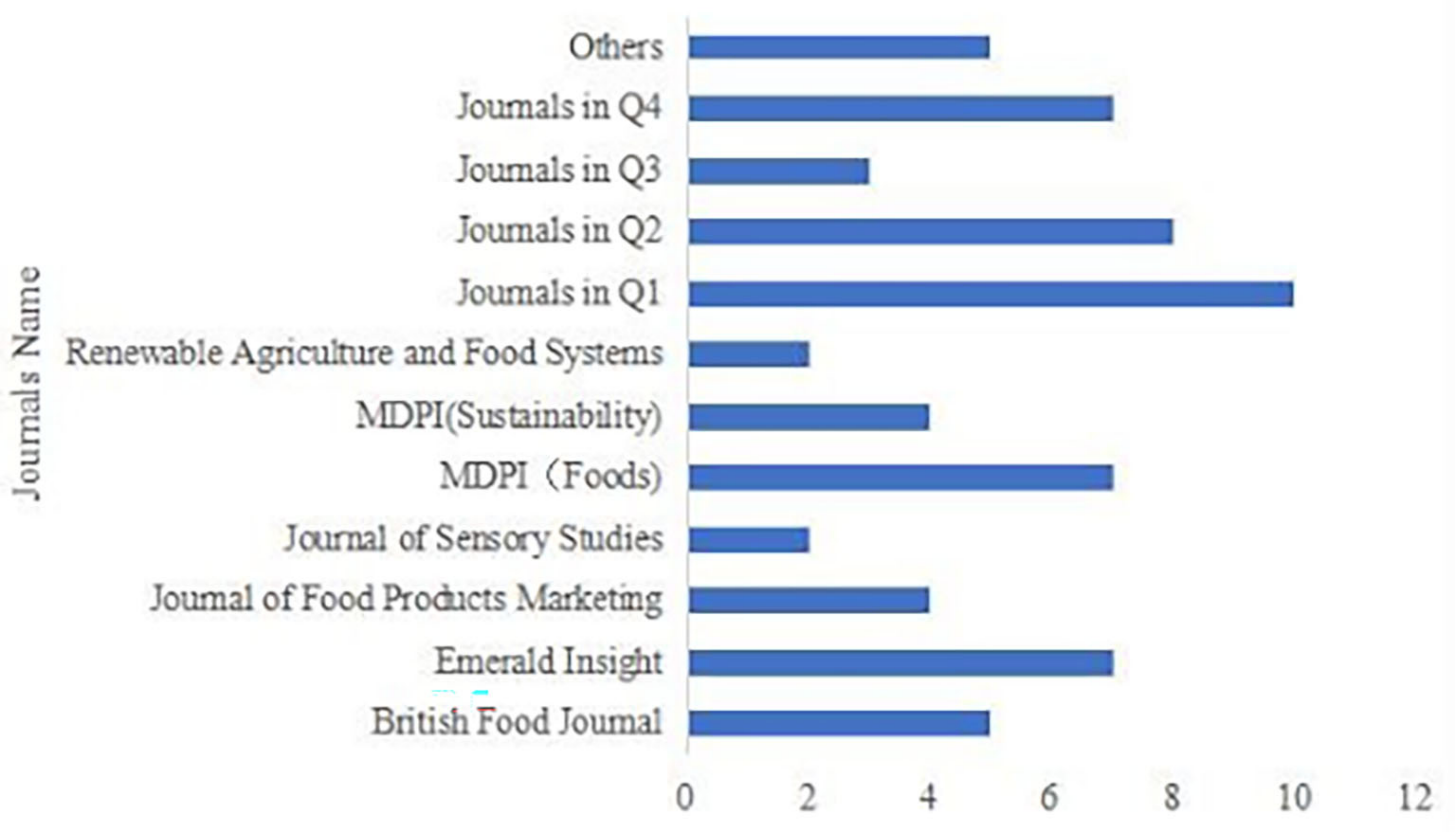
Journals distribution of publications.

These 64 articles were published in 40 different journals (see Figure 3).

Journal concentration was low, as 33 journals published only one article each. To provide a clearer overview, these journals were further grouped by Journal Impact Factor quartile (Q1, Q2, Q3, Q4, and others). The highest contributing outlets were MDPI Foods and Emerald Insight, each publishing seven articles. The British Food Journal published five articles, while the Journal of Food Products Marketing and MDPI Sustainability each published four. Most remaining journals published two or three articles at most.

*3.1.2 Article’s citation number*

Citation counts vary substantially across the included studies (see Appendix A), indicating a clear divide between highly cited and less cited articles. The most cited paper received 168 citations ( Gracia & de-Magistris, 2016), followed by 95 ( Marcoz, Melewar, & Dennis, 2016) and 60 citiations ( Bryla, 2017). These highly cited studies were published in well established outlets and primarily examine consumer attitudes and preferences toward food origin cues, as well as the role of certification and labelling in shaping perceived value.

In contrast, a number of studies have relatively low citation counts, typically between 0 and 10 citations ( Çukur, Kizilaslan, Kizilaslan, & Çukur, 2022; Nilgün-Doğan & Adanacıoğlu, 2022; Papoutsi, 2023). This pattern is likely related to shorter time since publication and differences in journal visibility. Therefore, citation counts in this review should be interpreted as an indicator of scholarly attention rather than as a direct measure of study quality.

### 3.2 Research design

*3.2.1 Study setting*

The included studies cover a wide range of countries and regions. As shown in Appendix B, Italy, Spain, Türkiye, and Serbia account for the largest number of consumer related studies on geographical indications. Italy contributes 26 studies (40%), followed by Spain with six studies (9%), Türkiye with five studies (7%), and Serbia with four studies (6%). The remaining studies are distributed across other countries, together accounting for 38% of the sample.

Across the 64 studies, data were collected using diverse approaches, including questionnaire surveys, interviews, online experiments, and in depth interviews. Several studies recruited participants through professional networking platforms or business email contacts, while others relied on survey platforms or online experimental tools. A smaller number of studies used observational approaches, semi structured interviews, or household scanning panel data.

The analytical techniques also varied. Common quantitative methods included regression-based models, factor analysis, cluster analysis, structural equation modelling, discrete choice experiments, and multilevel logistic regression. Research instruments included structured questionnaires and experimental designs such as experimental auctions, choice based experiments, and direct ranking tasks.

*3.2.2 Research methods*

Of the 64 articles, 48 employed quantitative designs, 11 used qualitative approaches, and five adopted mixed methods. Overall, the literature shows a strong preference for quantitative empirical designs when examining the relationship between geographical indications and consumer outcomes.

*3.2.3 Unit of sample*

The reviewed studies used different sampling units, which can be grouped into three broad categories: individuals, organizations, and specific target populations.

At the individual level, samples commonly consisted of general consumers and student participants. For example, several studies focused on Italian consumers (ID12, ID14, ID16, ID21, ID22, ID26, ID27, ID32, ID41, ID43, ID45, ID47, ID54, ID55, ID56, ID59, ID61, ID62, ID64), students in Brazil and Italy (ID2, ID5, ID10, ID18), and Spanish consumers (ID3, ID17, ID19, ID42, ID44, ID50).

At the organizational level, a smaller set of studies used firms or supply chain actors as the sampling unit, such as Italian companies (ID1, ID25) and producers and distributors in the Mediterranean area (ID20, ID31), including evidence drawn from Serbia related contexts.

In addition, some studies targeted specific consumer groups defined by product involvement or expertise. Examples include highly engaged consumers (ID33), Fontina cheese consumers in Italy (ID41), Brazilian coffee experts (ID13), and consumers of cheese, ham, and honey in Slovenia (ID19).

### 3.3 Theories

According to Zydev and Warner, theories can be coded into one of three types: grounded theoretical foundations, cited theoretical foundations, or theoretical foundations that are not provided ( Zydney & Warner, 2016).

*3.3.1 Grounded theoretical foundations*

Among the 64 papers, 25 (40%) provided clear statements about the theories used, as detailed in Appendix B. These theories cover multiple fields such as information economics, psychology, and behavioral science. This indicates that research on consumers of GIs agricultural products integrates the latest developments in economics, psychology, and behavioral science research.

*3.3.2 Cited theoretical foundations*

Sixteen papers (25%) mentioned or cited relevant theories but did not clearly operationalize them in the analysis of GI agricultural product consumption. The most frequently cited frameworks include the theory of planned behavior, value perception theory, and quality related theories. This indicates that many studies emphasize consumer intention, perceived value, and quality evaluation when discussing GI related consumption. Ethnocentrism theory is the second most frequently cited. Two papers (ID40 and ID50) draw on notions such as ethnic preference and geographic source identification to explain preferences for local products and traditional production methods, and to highlight geographic origin as a competitive signal, particularly for smaller producers. Other cited frameworks include hedonic theory (ID30).

*3.3.3 Theoretical foundations not provided*

Twenty-two papers (35%) did not report any theoretical framework to guide the study design or analysis.

### 3.4 Consumers responses and the determinants

Across the 64 articles, consumer responses to GI agri food products are reported in several forms. The most frequently examined responses are willingness to pay, preference, cognition, and purchase intention. Other responses include evaluation, actual consumption, perceived risk, attitude, and willingness to pay a premium, among others.

*3.4.1 Willingness to pay and the determinants*

Twelve studies examine willingness to pay. Most of them report a positive effect of GI related cues on consumers’ willingness to pay. In these studies, consumers are generally willing to pay more for products carrying GI, PDO, or PGI labels (ID3, ID5, ID8, ID18, ID21, ID27, ID34, ID41, ID50, ID64).

The determinants discussed in this stream can be grouped into three broad categories. First, consumer characteristics, such as sociodemographic profiles and involvement. Second, product and label attributes, including perceived quality, authenticity, and the presence of additional information on packaging. Third, market and context factors, such as price levels and the interaction between multiple labels.

Two studies report that GI labels do not increase willingness to pay in certain settings (ID15, ID47). For example, Papoutsi (2023) suggests that consumers value organic certification more strongly for olive oil, and adding multiple labels can reduce willingness to pay. Similarly, Stiletto and Trestini (2022) show that consumers are willing to pay more for products with Organic and Mountain Product logos regardless of PDO certification. When these attributes are combined with PDO, willingness to pay decreases, which suggests overlap or substitution between labels rather than simple additivity.

*3.4.2 Preferences and the determinants*

Eleven studies focus on consumer preferences (ID2, ID9, ID12, ID19, ID22, ID24, ID44, ID55, ID57, ID59, ID63). Several studies report that GI labelled products are more preferred than comparable alternatives ( Lambarraa-Lehnhardt, Ihle, and Elyoubi, 2021a; Rabadán, Martínez-Carrasco, Brugarolas, and Bernabéu. 2021; Skubic, Erjavec, and Klopcic. 2018a). Artêncio, Giraldi, and de Oliveira (2022) further shows heterogeneity by gender, where women are more sensitive to origin information, while men show stronger preferences for GI coffee.

Across this group, determinants of preferences include sociodemographic characteristics, consumer involvement, and product attributes. Frequently mentioned product cues include sensory attributes such as taste and freshness, origin distance, production methods, price, branding, and in some cases specific taste profiles such as spiciness.

*3.4.3 Purchase intention and the determinants*

Eleven studies examine purchase intention (ID4, ID14, ID17, ID23, ID26, ID33, ID38, ID45, ID49, ID51, ID61). Overall, these studies suggest that GI labels can increase purchase intention by improving perceived quality and strengthening trust. Several articles show that consumers’ understanding of GI and origin information can increase trust and satisfaction (ID14, ID17). Other studies stress the role of perceived uniqueness and quality assurance associated with GI certification (ID33). Place related factors also appear important. For example, place attachment and identification with traditional production methods can increase willingness and purchase frequency (ID23). One study further highlights solidarity related motivations, where identification with earthquake affected areas supports supportive buying behaviour (ID61). However, insufficient understanding of certified labels can weaken purchase intention, as reported for Greek consumers (ID4).

The determinants reported in this stream include origin cues, labelling and health claims, trust, perceived quality, attitudes, subjective norms, perceived behavioural control, transparency of information, place attachment, sense of belonging, and sociodemographic characteristics.

*3.4.4 Others and the determinants*

The remaining studies cover a wider set of outcomes (Table 4). Some outcomes are examined in only one study, such as loyalty (ID1), ethnocentrism (ID32), perception and evaluation (ID40), and word of mouth communication (ID58). A small number of studies focus on actual consumption (ID6, ID7), attitudes (ID16, ID25, ID28), consumer participation (ID25, ID31), price perceptions (ID30, ID43, ID62), and willingness to pay additional fees (ID29, ID39). Overall, evidence on these outcomes is more fragmented than for willingness to pay, preference, and purchase intention.

### 3.5 Information and effect

*3.5.1 Related information mentioned*

The reviewed studies consistently indicate that GI related information influences consumers. The most frequently discussed information concerns product related cues, reported in 19 studies (ID2, ID4, ID5, ID8, ID10, ID17, ID25, ID26, ID27, ID33, ID35, ID36, ID37, ID38, ID42, ID43, ID56, ID57, ID60). These cues include packaging (ID2), product quality (ID26, ID37, ID42, ID57), and intrinsic attributes such as appearance, colour, and taste (ID17, ID36, ID37, ID56). Several studies also highlight production methods and business culture as relevant information (ID8, ID25, ID27, ID37, ID38, ID43). In addition, advertising is mentioned in one study (ID10), and narrative-based information is noted in another (ID11).

Labelling is the second most frequently discussed information category, appearing in ten studies (ID2, ID3, ID13, ID14, ID15, ID26, ID42, ID44, ID54, ID55). Certifications by official bodies are also highlighted in seven studies (ID3, ID15, ID33, ID35, ID41, ID47, ID56), indicating that institutional endorsement is an important cue in consumer evaluation.

*3.5.2 The effect on consumers*

Across the sample, most studies report positive effects of GI related information on consumers. Many articles indicate higher willingness to pay a premium and stronger purchase related responses when GI cues are present, often because GI labels increase perceived local distinctiveness, improve perceived quality, and reduce information uncertainty (e.g., ID3, ID5, ID14, ID26, ID36, ID43, ID47).

A second consistent effect is the strengthening of consumer perceptions, trust, and satisfaction. Several studies suggest that clear GI and origin information helps consumers recognise products, reduces perceived risk, and increases confidence in product reliability and quality (e.g., ID2, ID4, ID10, ID14, ID17, ID33, ID38).

Effects also vary by consumer characteristics. For example, gender differences are reported in sensitivity to origin cues (ID2). Some studies report differences by age, gender, education, or expertise, especially for sensory evaluation (ID11, ID13).

Negative or mixed effects are also reported in a smaller subset of studies. For example, organic or other quality labels may dominate consumer valuation and reduce the marginal value of PDO certification (ID15, ID47). High prices can also lead to utility losses for GI products in certain markets (ID35). One study suggests that introducing quality labelling can reduce the perceived quality of other GI products, implying potential spillover effects within a category (ID54). In addition, one study reports that for Slovenian consumers, product quality and health impacts are more important than origin labels (ID46).

## 4. Discussion

### 4.1 Research trends

This review shows a clear increase in publications after 2021, suggesting that consumer research on GI agri food products is gaining momentum. At the same time, the evidence base remains dispersed across outlets and contexts. The 64 studies are published in 40 journals, and most journals contribute only one article. This dispersion indicates that the topic is still cross disciplinary and has not yet formed a stable core journal community. The citation pattern also reflects this structure. A small number of widely cited studies focus on origin cues and certification related consumer valuation, while many newer studies have low citation counts, which is expected given the time needed for citations to accumulate.

A second trend is the strong geographical concentration. Italy accounts for 40 percent of the included studies, followed by Spain, Türkiye, and Serbia. This concentration helps explain why the literature often emphasizes European certification schemes and familiar product categories. It also limits generalizability to broader settings. This observation is consistent with the concern raised in prior reviews that evidence is uneven across contexts. For instance, Thøgersen (2023) synthesizes consumer choice studies largely from OECD countries, while other reviews either focus on specific regions or on selected countries. Our results confirm that, even within the consumer-focused literature, the distribution of study settings is still unbalanced. This implies that many determinants identified in the literature may be context dependent, especially where the institutional credibility of GI systems and consumer familiarity differ.

A third trend relates to research design and theory use. Quantitative designs dominate the field, with fewer qualitative or mixed methods studies. This supports the idea that the field prioritizes measurable consumer outcomes such as willingness to pay and purchase intention. However, theory use is uneven. Only 40 percent of studies provide grounded theoretical foundations, while a sizeable group either cites theories without clear application or provides no explicit theory. This pattern suggests that the literature has strong empirical activity but weaker cumulative theory building, which makes it harder to compare findings across products and contexts.

### 4.2 Consumers responses and determinants

Across the reviewed studies, willingness to pay, preference, and purchase intention are the most frequently examined responses, and the overall direction is positive. This aligns with the broad conclusion reported in earlier consumer-oriented syntheses. For example, Thøgersen (2023) shows that origin related cues often shape consumer evaluation and choice, and our results specify how this plays out in GI agri food products through outcomes that are directly linked to market behavior, such as willingness to pay and purchase intention. At the same time, the evidence also shows clear heterogeneity, which helps explain why results are not always consistent across studies.

First, several studies indicate that GI labels work as signals that reduce uncertainty and strengthen perceived quality and authenticity. This mechanism is reflected in repeated findings that GI cues increase trust and perceived value, which then support higher willingness to pay and stronger purchase intention. The determinants reported in the literature can be summarized into three levels. Consumer level factors include sociodemographic characteristics and involvement. Product level factors include sensory and intrinsic attributes such as taste and freshness. Information and system level factors include trust in labels, perceived transparency, and certification credibility.

Second, the review identifies conditions under which GI cues do not increase willingness to pay and may even reduce it. These findings suggest that the effectiveness of GI labels depends on the information environment and competing signals. In particular, organic labels and other quality cues can substitute for GI cues. When multiple labels are introduced, the marginal value of GI certification can decline. This provides a concrete explanation for mixed results reported in some studies and shows that consumer valuation is not simply additive across labels.

Third, place-based motivations appear as an important pathway in the purchase intention literature. Findings related to place attachment, social identity, and solidarity purchasing indicate that GI products can also trigger symbolic and social meanings beyond quality evaluation. This complements purely economic explanations and supports the interdisciplinary nature of the field. However, because these mechanisms are discussed in fewer studies, they remain less consolidated than the quality and trust pathway.

Overall, our synthesis extends earlier reviews that focused on a single outcome or a single actor. For example, Deselnicu et al. (2013) provides a quantitative summary of price premiums, but our results show that willingness to pay is shaped by broader determinants, including information transparency, authenticity perceptions, and label interactions. Similarly, Marion, Luisa, and Sebastian (2023) focus on GI adoption by small and medium enterprises, whereas our findings clarify the consumer side responses that partly determine whether such adoption translates into market value.

### 4.3 Information types and effects

The evidence indicates that consumers respond to multiple types of GI related information. Product related cues are the most frequently discussed, including packaging, perceived quality, and intrinsic attributes such as appearance, colour, and taste. Labelling cues and official certification cues are also repeatedly highlighted. This pattern supports the view that GI effects are not driven by the label alone. Instead, GI cues often operate together with other information, and consumer responses depend on how these cues are presented and interpreted.

Most studies report positive effects. GI related information tends to increase trust, perceived quality, and satisfaction, and it often increases willingness to pay a premium. These findings are consistent with the consumer perception focus reported in Glogovețan et al. (2022), which highlights that awareness and understanding of certification labels are central to consumer evaluation. Our review adds that these perception effects frequently translate into behavioural outcomes, especially purchase intention and willingness to pay, when labels are supported by credible certification and clear product information.

At the same time, the review also documents negative and mixed effects in a smaller number of studies. These cases are important because they clarify boundary conditions. Negative effects are more likely when GI cues compete with stronger cues, such as organic certification, when high prices reduce consumer utility, or when additional labelling creates unintended spillovers that affect perceived quality of other GI products. The Slovenian evidence that quality and health impacts outweigh origin labels also indicates that consumers may prioritise different information depending on local consumption norms and market conditions. These patterns suggest that GI information works best when it is credible, easy to interpret, and not overshadowed by competing signals.

Taken together, the discussion highlights an evidence base that is growing but still fragmented by geography, methods, and theory use. The consumer side effects of GI information are generally positive, but they are not universal. Future research can improve comparability by reporting clearer theoretical frameworks, testing label interaction effects more systematically, and expanding evidence beyond the most studied countries and product categories.

## 5. Conclusion

In conclusion, this systematic review analyzes evidence on the determinants of consumption of geographically indicated agricultural products and the consumer responses associated with these determinants. The findings reflect growing scholarly attention to geographical indications and suggest that GI schemes can support value creation for agri food products by shaping consumer perceptions and purchase related outcomes. At the same time, the review identifies clear gaps in the current literature. Existing studies remain uneven in their focus on consumption determinants and are geographically concentrated, which limits the comparability and generalizability of findings across contexts. Future research should therefore expand cross national evidence and examine consumer behavior, preferences, and decision making in more diverse settings. In addition, accurate and transparent GI related information is essential to strengthen consumer trust and reduce confusion, especially in markets where multiple labels coexist.

Overall, a clearer understanding of consumer responses and more effective information communication can support the design and implementation of GI schemes and enhance their role in agricultural food markets.

Ethical & consent: Ethical approval and consent were not required.

## Data Availability

No data are associated with this article Figshare: Flow of PRISMA.jpg, https://doi.org/10.6084/m9.figshare.27627237.v1 ( Tan, 2024b). Data are available under the terms of the CC0 1.0 Universal. Figshare: Dataset.PRISMA checklist, https://doi.org/10.6084/m9.figshare.27627324.v1 ( Tan, 2024a). Data are available under the terms of the CC0 1.0 Universal. Figshare: Consumers responses and determinants, Details of the reviewed studies, List of theoretical foundations and Methodologies cited in reviewed studies, https://doi.org/10.6084/m9.figshare.27627480.v1 ( Tan, 2024c). The project contains following data:
1.
Table 4, 2. Appendix A, 3. Apeendix B Data are available under the terms of the CC0 1.0 Universal.

## Appendix A. Details of the reviewed studies.

**Table:**

ID	Title	Journal of Publication	Publication Year	Total Citations
ID1	Marketing geographical indication products in the digital age: a holistic perspective ( Bartoli, Bonetti, & Mattiacci, 2022)	British Food Journal	2022	4
ID2	A cup of black coffee with GI, please! Evidence of geographical indication influence on a coffee tasting experiment ( Artêncio et al., 2022)	Physiology & Behavior	2022	5
ID3	Labels for a Local Food Speciality Product: The Case of Saffron ( Sanjuan-Lopez & Resano-Ezcaray, 2020)	Journal of Agricultural Economics	2020	24
ID4	Consumers’ intention to buy protected designation of origin and protected geographical indication foodstuffs: the case of Greece ( Likoudis, Sdrali, Costarelli, & Apostolopoulos, 2016)	International Journal of Consumer Studies	2016	27
ID5	Brand Coopetition with Geographical Indications: Which Information Does Lead to Brand Differentiation? ( Dentoni, Tonsor, Calantone, & Peterson, 2013)	New Medit	2013	16
ID6	AN ANALYSIS OF THE FACTORS AFFECTING THE CONSUMPTION OF GEOGRAPHICALLY INDICATED PRODUCTS USING DECISION TREE AND ARTIFICIAL NEURAL NETWORKS ( Çukur et al., 2022)	Journal of Animal & Plant Sciences	2022	N/A
ID7	CONSUMERS PERCEPTION AND BEHAVIOUR TOWARDS GEOGRAPHICAL INDICATION PRODUCTS: THE CASE OF TRADITIONAL PESTIL FROM GUMUSHANE, TURKEY ( Nilgün-Doğan & Adanacıoğlu, 2022)	Agrociencia	2022	N/A
ID8	Consumers’ Preferences for the Consumption of the Fresh Black Slavonian Pig’s Meat ( Jelić Milković et al., 2023)	MDPI (Foods)	2023	4
ID9	How Successful Is Origin Labeling in a Developing Country Context? Moroccan Consumers’ Preferences toward Local Products ( Lambarraa-Lehnhardt, Ihle, & Elyoubi, 2021a)	MDPI (Sustainability)	2021	21
ID10	Product versus region of origin: which wins in consumer persuasion? ( Luceri, Latusi, & Zerbini, 2016)	Emerald Insight	2016	51
ID11	The impact of coffee origin information on sensory and hedonic judgment of fine Amazonian robusta coffee ( Artencio et al., 2023)	Journal of Sensory Studies	2022	2
ID12	Study of Consumer Preferences in Regard to the Blonde Orange Cv. Washington Navel “Arancia Di Ribera PDO” ( Ingrassia, Sgroi, Tudisca, & Chironi, 2017)	Journal of Food Products Marketing	2016	26
ID13	Consumers’ hedonic liking of different labeled and conventional food products in Slovenia ( Skubic, Erjavec, Ule, & Klopcic, 2018b)	Journal of Sensory Studies	2018	17
ID14	Familiar worldwide: how PDO products reflect quality in consumers’ appraisal and behaviour ( Toma, Manta, Morrone, & Campobasso, 2023)	Emerald Insight	2022	6
ID15	Consumer Valuation of European Certification Labels on Extra Virgin Olive Oil: Assessing the Impact of Multiple Labels and Consumer Heterogeneity ( Papoutsi, 2023)	Journal of Food Products Marketing	2023	N/A
ID16	The Value of Region of Origin, Producer and Protected Designation of Origin Label for Visitors and Locals: The Case of Fontina Cheese in Italy ( Marcoz et al., 2016)	International Journal of Tourism Research	2016	95
ID17	Exploring the mediating role of trust in food products with Protected Designation of Origin. The case of “Jamón de Teruel” ( Fandos-Herrera, 2016)	Spanish Journal of Agricultural Research	2016	15
ID18	The effect of information and co-branding strategies on consumers willingness to pay (WTP) for Protected Designation of Origin (PDO) products: the case of pre-sliced Parma Ham ( Arfini & Mancini, 2015)	Progress in Nutrition	2015	11
ID19	Perceptions of geographical indication labels as quality indicators inside and outside the labels’ area of influence: the case of spring fruits ( Rabadán et al., 2021)	Renewable Agriculture and Food Systems	2021	10
ID20	Market-Oriented Sustainability of Sjenica Sheep Cheese ( Filipovic, 2019)	MDPI (Sustainability)	2019	4
ID21	Development and Validation of the Perceived Authenticity Scale for Cheese Specialties with Protected Designation of Origin ( Sidali, Capitello, & Manurung, 2021)	MDPI (Foods)	2021	9
ID22	Understanding the Role of Purchasing Predictors in the Consumer’s Preferences for PDO Labelled Honey ( Di Vita, Pippinato, et al., 2021a)	Journal of Food Products Marketing	2021	19
ID23	The effect of place attachment of geographical indication agricultural products on repurchase intention ( Zhe, Jie, & Yuan, 2023)	Journal of Retailing and Consumer Services	2023	7
ID24	Consumer preferences regarding national and EU quality labels for cheese, ham and honey The case of Slovenia ( Skubic, Erjavec, & Klopcic, 2018a)	Emerald Insight	2017	53
ID25	Communication patterns to address the consumption of PDO products ( Bonetti, Mattiacci, & Simoni, 2019)	Emerald Insight	2019	6
ID26	Choice Drivers for Quality-Labelled Food: A Cross-Cultural Comparison on PDO Cheese ( Menozzi, Giraud, Saïdi, & Yeh, 2021)	MDPI (Foods)	2021	2
ID27	Do consumers really recognise a distinct quality hierarchy amongst PDO sparkling wines? The answer from experimental auctions ( Galletto et al., 2021)	Emerald Insight	2020	11
ID28	CONSUMER ATTITUDES AND HABITS ABOUT PRODUCTS WITH GEOGRAPHICAL INDICATION IN SERBIA ( Ciric, Ciric, Pivac, & Besermenji, 2023)	Економика пољопривредеЕкономика пољопривреде	2023	N/A
ID29	Do South African Consumers have an Appetite for an Originbased Certification System for Meat Products? A Synthesis of Studies on Perceptions, Preferences and Experiments ( Kirsten et al., 2017)	International Journal on Food System Dynamics	2017	15
ID30	Valuing country of origin and organic claim A hedonic analysis of cheese purchases of German households ( Schröck, 2014)	Emerald Insight	2013	48
ID31	Establishing Communities of Value for Sustainable Localized Food Products: The Case of Mediterranean Olive Oil ( Radić, Monaco, Cerdan, & Peri, 2023)	MDPI (Sustainability)	2023	1
ID32	Where was my cup of honey made? PDO honey and sub-regional ethnocentric consumer segments ( Trentinaglia, Cavicchioli, Pocol, & Baldi, 2023)	Emerald Insight	2022	1
ID33	Drivers of high-involvement consumers’ intention to buy PDOwines: Valpolicella PDO case study ( Capitello, Agnoli, & Begalli, 2016)	Journal of the Science of Food and Agriculture	2015	12
ID34	Consumers’ willingness to pay for geographical origin labels: evidence from the Turkish table olive ( Ozkan & Gurbuz, 2023)	researchgate	2022	N/A
ID35	Consumers’ Valuation of Geographical Indication-Labeled Food: The Case of Hom Mali Rice in Bangkok ( Lee, Pavasopon, Napasintuwong, & Nayga, 2020)	Asian Economic Journal	2020	9
ID36	Consumers’ Preference for the Consumption of the Fresh Black Slavonian Pig’s Meat ( Milkovic et al., 2023)	MDPI (Foods)	2023	4
ID37	The Role of GI Products or Local Products in the Environment—Consumer Awareness and Preferences in Albania, Bulgaria and Poland ( Muça, Pomianek, & Peneva, 2022)	MDPI (Sustainability)	2021	21
ID38	Do consumers intend to purchase the food with Geographical Indication? ( Aytop & Cankaya, 2022)	Mediterranean journal of economics, agriculture and environment	2022	2
ID39	MARKET PERSPECTIVES FOR SERBIAN PDO PRODUCTS IN THE REPUBLIC OF SERBIA ( Panin, 2022)	THE CENTRAL EUROPEAN JOURNAL OF REGIONAL DEVELOPMENT AND TOURISM	2022	N/A
ID40	THE EFFECT OF A COUNTRY NAME ON CONSUMERS’ PERCEPTION AND ASSESSMENT OF AGRICULTURAL PRODUCTS WITH PROTECTED DESIGNATION OF ORIGIN ( Rakic, Bugarcic, & Draskovic, 2022)	Economics of Agriculture	2022	1
ID41	Willingness to pay for P.D.O. certification: an empirical investigation ( Garavaglia & Marcoz, 2014)	Int. J. Food System Dynamics	2014	1
ID42	Not Seeing the Forest for the Trees: The Impact of Multiple Labelling on Consumer Choices for Olive Oil ( Pérez, Gracia, & Barreiro-Hurlé, 2020)	MDPI (Foods)	2020	20
ID43	Consumer Preferences for Cheese Products with Quality Labels: The Case of Parmigiano Reggiano and Comte ( Menozzi, Yeh, Cozzi, & Arfini, 2022)	MDPI (Animals)	2022	6
ID44	Consumer preferences for food labeling: What ranks first ( Gracia & de-Magistris, 2016)	Food Control	2016	168
ID45	An investigation into Italian consumers’ awareness, perception, knowledge of European Union quality certifications, and consumption of agri-food products carrying those certifications ( Sampalean, Rama, & Visentin, 2021)	Bio-based and Applied Economics Journal	2021	6
ID46	Consumer awareness of PDO-labelled food in Slovenia ( Skubic, Erjavec, & Klopcic, 2019)	Italian Journal of Animal Science	2019	18
ID47	When less isn’t more and more isn’t less: is there an overlap between “protected designation of origin”, “mountain product” and “organic” in Italy? ( Stiletto & Trestini, 2022)	British Food Journal	2023	4
ID48	Consumers’ awareness of the EU’s protected designations of origin logo ( Goudis & Skuras, 2021)	British Food Journal	2021	29
ID49	Purchasing local and non-local products labeled with geographical indications (GIs) ( Albayram, Mattas, & Tsakiridou, 2014)	Operational Research	2014	20
ID50	Geographical indication food products and ethnocentric tendencies: The importance of proximity, tradition, and ethnicity ( Fernández-Ferrín et al., 2019)	Journal of Cleaner Production	2019	57
ID51	Foreign Geographical Indications, Consumer Preferences, and the Domestic Market for Cheese ( Slade, Michler, & Josephson, 2019)	Applied Economic Perspectives and Policy	2019	28
ID52	Geographical indications for supporting rural development in the context of the Green Morocco Plan: Oasis dates ( Lambarraa-Lehnhardt, Ihle, & Mhaouch, 2021b)	Agricultural Economics/Zemědělská Ekonomika	2021	7
ID53	The perception of EU quality signs for origin and organic food products among Polish consumer ( Bryla, 2017)	Quality Assurance and Safety of Crops & Foods	2017	60
ID54	Vertical differentiation via multi-tier geographical indications and the consumer perception of quality: The case of Chianti wines ( Costanigro, Scozzafava, & Casini, 2019)	Food Policy	2019	24
ID55	EU quality labels in the Italian olive oil market: How much overlap is there between geographical indication and organic production? ( Roselli, Giannoccaro, Carlucci, & De Gennaro, 2018)	Journal of Food Products Marketing	2018	29
ID56	Italian consumers’ awareness, preferences and attitudes about Sicilian blood oranges (Arancia Rossa di Sicilia PGI) ( Selvaggi, Zarbà, Pappalardo, Pecorino, & Chinnici, 2023)	Journal of Agriculture and Food Research	2023	6
ID57	Food traditions and consumer preferences for cured meats: Role of information in geographical indications ( Sgroi, 2021)	International Journal of Gastronomy and Food Science	2021	17
ID58	Geographical indication labelling of food and behavioural intentions ( Chen, 2021)	British Food Journal	2021	7
ID59	Oh my darling clementine: heterogeneous preferences for sustainable citrus fruits ( Di Vita, Vecchio, et al., 2021b)	Renewable Agriculture and Food Systems	2021	12
ID60	Effect of Geographical Indication Information on Consumer Acceptability of Cooked Aromatic Rice ( Arroyo, Hogan, Wisdom, Moldenhauer, & Seo, 2020)	MDPI (Foods)	2020	12
ID61	May trust and solidarity defy food scares? The case of Parmigiano-Reggiano PDO sales in the aftermath of natural disaster ( Menozzi & Finardi, 2019)	British Food Journal	2019	13
ID62	How Much Do Consumers Value Protected Designation of Origin Certifications? Estimates of willingness to Pay for PDO Dry-Cured Ham in Italy ( Garavaglia & Mariani, 2017)	Agribusiness	2017	54
ID63	Understanding Consumers’ Preferences for Protected Geographical Indications: A Choice Experiment with Hungarian Sausage Consumers ( Török, Gorton, Yeh, Czine, & Balogh, 2022)	MDPI (Foods)	2022	18
ID64	Consumer awareness of sustainable supply chains: A choice experiment on Parma ham PDO ( Mazzocchi, Orsi, Zilia, Costantini, & Bacenetti, 2022)	Science of The Total Environment	2022	11
