# Supplementary material for: Consumer responses and determinants in geographical indications agricultural product consumption: A ten-year systematic review
Source: F1000Res. 2026 Apr 15;13:1410. Originally published 2024 Nov 22. [Version 3] doi: 10.12688/f1000research.158225.3 (PMC13129523; doi:10.12688/f1000research.158225.3)
Supplement: Peer review history

# Reviewer report: Reviewer response for version 3

**DOI:** 10.5256/f1000research.197729.r475864

**Published:** 2026-05-09

**Authors:** Patricia Manzano Fischer

**Affiliations:** 1 Department of Food Sciences, Universidad Autónoma Metropolitana, Mexico City, Mexico, Mexico; 2 Universidad Nacional Autónoma de México, Mexico City, Mexico

The article presents a comprehensive systematic review of consumer responses and determinants in the consumption of geographical indication agricultural products. The rationale and objectives are clearly stated.

 There are sufficient details regarding the methods and analysis to allow replication by other researchers. The study reviews a ten-year period of peer-reviewed journal articles published in English and focuses on four European countries. These limitations (English only articles reviewed and lack of other countries context) are clearly acknowledged and presented as an opportunity for future research.

 Although no statistical analysis was conducted, the review findings are adequately analyzed and interpreted.

 The discussion is well structured and reflects the recommendations of the previous reviewer. The article now includes a clear conceptual framework, incorporating a three-level determinant structure and stronger theoretical integration.

 The conclusions are adequately supported by the results. The authors have also strengthened the conclusion following the suggestions of a previous reviewer.

 The authors have stated that no Living Systematic Review methodology was adopted in their work.

 The authors have addressed the previous reviewers’ comments, and the manuscript has improved substantially. I consider the article ready for indexing.

Are the rationale for, and objectives of, the Systematic Review clearly stated?

Yes

Is the statistical analysis and its interpretation appropriate?

Not applicable

If this is a Living Systematic Review, is the ‘living’ method appropriate and is the search schedule clearly defined and justified? (‘Living Systematic Review’ or a variation of this term should be included in the title.)

Not applicable

Are sufficient details of the methods and analysis provided to allow replication by others?

Yes

Are the conclusions drawn adequately supported by the results presented in the review?

Yes

Reviewer Expertise:

Food sustainability and behavioral change

I confirm that I have read this submission and believe that I have an appropriate level of expertise to confirm that it is of an acceptable scientific standard.

# Reviewer report: Reviewer response for version 3

**DOI:** 10.5256/f1000research.197729.r475373

**Published:** 2026-04-29

**Authors:** Pandu Laksono

**Affiliations:** 1 National Research and Innovation Agency, Jakarta, Indonesia

The authors have satisfactorily addressed the comments and revised the manuscript accordingly. I am satisfied with the improvements and responses provided. Therefore, I recommend that this paper be **approved **for indexing.

Are the rationale for, and objectives of, the Systematic Review clearly stated?

Yes

Is the statistical analysis and its interpretation appropriate?

Not applicable

If this is a Living Systematic Review, is the ‘living’ method appropriate and is the search schedule clearly defined and justified? (‘Living Systematic Review’ or a variation of this term should be included in the title.)

No

Are sufficient details of the methods and analysis provided to allow replication by others?

Yes

Are the conclusions drawn adequately supported by the results presented in the review?

Partly

Reviewer Expertise:

Behavioral Economics, Agricultural Economics

I confirm that I have read this submission and believe that I have an appropriate level of expertise to confirm that it is of an acceptable scientific standard.

# Reviewer report: Reviewer response for version 3

**DOI:** 10.5256/f1000research.197729.r475372

**Published:** 2026-04-22

**Authors:** Nilgun Dogan

**Affiliations:** 1 Gümüşhane University, Gümüşhane, Turkey

I have no further comments to make.

Are the rationale for, and objectives of, the Systematic Review clearly stated?

Yes

Is the statistical analysis and its interpretation appropriate?

Partly

If this is a Living Systematic Review, is the ‘living’ method appropriate and is the search schedule clearly defined and justified? (‘Living Systematic Review’ or a variation of this term should be included in the title.)

Yes

Are sufficient details of the methods and analysis provided to allow replication by others?

Partly

Are the conclusions drawn adequately supported by the results presented in the review?

Partly

Reviewer Expertise:

agricultural economy

I confirm that I have read this submission and believe that I have an appropriate level of expertise to confirm that it is of an acceptable scientific standard.

# Reviewer report: Reviewer response for version 2

**DOI:** 10.5256/f1000research.195125.r455281

**Published:** 2026-02-28

**Authors:** Pandu Laksono

**Affiliations:** 1 National Research and Innovation Agency, Jakarta, Indonesia

This manuscript presents a PRISMA-guided systematic review of consumer responses and determinants in the context of geographical indication (GI) agricultural products over the period 2013–2023. 

 The manuscript demonstrates clear effort in organizing the literature and provides a structured synthesis of 64 empirical studies. However, while the study is informative, several methodological and analytical aspects require strengthening to meet a higher scientific standard.

 Below I provide detailed comments:

1. Although the manuscript summarizes findings across 64 studies, the synthesis remains largely descriptive. The Results and Discussion sections tend to report patterns (e.g., “most studies show positive effects”) without sufficiently comparing effect magnitudes across contexts, explaining inconsistencies systematically and identifying causal mechanisms. The discussion would benefit from a conceptual framework summarizing the determinant structure (consumer-level, product-level, system-level factors), clearer theoretical integration (e.g., signalling theory, information asymmetry, TPB, trust models) and dentification of unresolved contradictions in the literature.
2. Please see section 3.3.3. There is information about 35% of the included studies lack explicit theoretical foundations. However, the manuscript stops at reporting this statistic.
3. See section 3.2.1, the heavy concentration of studies in Italy (40%) is acknowledged, but the implications are not sufficiently developed. Are determinants stable across institutional context? How does trust in certification differ between EU and non-EU countries? I think a stronger comparative discussion would improve the scientific contribution.
4. The review doesn’t include any methodological quality assessment on include studies and risk-of-bias evaluation.
5. The manuscript does not implement a Living Systematic Review methodology (single search date: 22 December 2023. This is acceptable, but the manuscript should ensure no ambiguity regarding this.
6. Some minor issues could be fixed,
  - Some sections remain repetitive between Results and Discussion.
  - Language has improved, but minor phrasing refinements could still enhance clarity.
  - Tables are informative but could benefit from more analytical summarization.
  - The conclusion is somewhat general and could be more forward-looking.

Overall, the manuscript makes a valuable contribution by consolidating a decade of research on consumer responses to GI agricultural products and by clarifying dominant research trends and outcome categories. With some further refinement in theoretical integration, analytical depth, and reflective discussion of methodological robustness and contextual variation, the study would offer an even stronger and more impactful synthesis of the literature.

 In conclusion, I find the manuscript suitable for indexing in principle, subject to moderate revision. The topic is important, the methodology is transparent, and the evidence base is clearly organized. I therefore recommend revision, with particular attention to strengthening theoretical synthesis and deepening the analytical discussion. With these improvements, the manuscript has strong potential to serve as a useful reference for researchers and policymakers working on geographical indications and consumer behaviour.

Are the rationale for, and objectives of, the Systematic Review clearly stated?

Yes

Is the statistical analysis and its interpretation appropriate?

Not applicable

If this is a Living Systematic Review, is the ‘living’ method appropriate and is the search schedule clearly defined and justified? (‘Living Systematic Review’ or a variation of this term should be included in the title.)

No

Are sufficient details of the methods and analysis provided to allow replication by others?

Yes

Are the conclusions drawn adequately supported by the results presented in the review?

Partly

Reviewer Expertise:

Behavioral Economics, Agricultural Economics

I confirm that I have read this submission and believe that I have an appropriate level of expertise to confirm that it is of an acceptable scientific standard, however I have significant reservations, as outlined above.

# Response

**Published:** 2026-03-13

**Authors:** AILIN TAN

**Affiliations:** Faculty of Economic and Manage, National University of Malaysia Faculty of Economic and Management, Bangi, Selangor, Malaysia

**Comment 1:** *The synthesis remains largely descriptive. The Results and Discussion sections tend to report patterns without sufficiently comparing effect magnitudes across contexts, explaining inconsistencies systematically, or identifying causal mechanisms. The discussion would benefit from a conceptual framework summarizing the determinant structure, clearer theoretical integration, and identification of unresolved contradictions.*

**Response:** We thank the reviewer for this central observation. We have made three substantive revisions in response.

 First, we have added a conceptual framework figure (Figure 4, inserted in Section 4.2) that visually organises the three-level determinant structure — consumer-level, product-level, and system-level factors — and maps these to their respective theoretical foundations and consumer response outcomes.

 Second, we have substantially deepened the theoretical integration in Section 4.2. A new paragraph now explicitly anchors each determinant level within complementary frameworks: signalling theory and information asymmetry models (Akerlof, 1970; Spence, 1973) explain how GI certification reduces the producer–consumer information gap at the system level; the Theory of Planned Behaviour (TPB) accounts for the joint role of attitudes, subjective norms, and perceived behavioural control at the consumer level; and consumer trust models explain how intrinsic and extrinsic product cues interact to shape overall product evaluation at the product level.

 Third, we have revised the synthesis paragraph at the close of Section 4.3 to explicitly identify three unresolved contradictions in the literature: (1) the conditions under which GI labels add value versus when they are substituted or crowded out by competing quality signals; (2) whether EU-derived determinants generalise to non-EU institutional contexts; and (3) the independent contribution of place-based and social-identity mechanisms to purchase decisions. Specific future research directions — longitudinal designs, cross-national comparisons, and experimental approaches — are now proposed.

**Comment 2:** *Section 3.3.3 reports that 35% of included studies lack explicit theoretical foundations but stops at reporting this statistic.*

**Response:** Accepted. We have expanded Section 3.3.3 with a new paragraph discussing the implications of this finding. We explain that atheoretical studies tend to report findings in isolation, limiting cross-context comparability, the ability to reconcile contradictions, and the identification of causal mechanisms. We also call explicitly on future research to ground empirical work within established frameworks — including signalling theory, information asymmetry models, TPB, and consumer trust models — to strengthen the theoretical coherence of this literature.

**Comment 3:** *The heavy concentration of studies in Italy (40%) is acknowledged but insufficiently developed. Are determinants stable across institutional contexts? How does trust in certification differ between EU and non-EU countries?*

**Response:** Accepted. We have substantially expanded the discussion of geographical concentration in Section 3.2.1. A new passage now contrasts the mature EU GI governance environment — characterised by well-established regulatory infrastructure, high consumer familiarity with PDO/PGI labels, and strong institutional credibility — with non-EU contexts such as Turkey, China, and emerging economies, where GI schemes operate under different regulatory frameworks with varying levels of consumer awareness and enforcement capacity. We argue that key determinants such as certification trust, label comprehension, and perceived authenticity may function differently across these environments, and we call for future research to explicitly test whether the three-level determinant structure identified in this review holds across divergent institutional settings.

**Comment 4:** *The review does not include any methodological quality assessment or risk-of-bias evaluation.*

**Response:** Accepted. We acknowledge this as a genuine limitation and have added a new paragraph in Section 4.1 (Discussion) that explicitly addresses this gap. We note that no standardised risk-of-bias instrument — such as the Mixed Methods Appraisal Tool (MMAT) or the Newcastle-Ottawa Scale — was applied to evaluate the internal validity of individual studies, and we explain why this is a common challenge in consumer behaviour systematic reviews given the heterogeneity of study designs. We caution readers accordingly and recommend that future systematic reviews in this domain incorporate formal quality appraisal protocols.

**Comment 5:** *The manuscript does not implement a Living Systematic Review methodology, which is acceptable, but the manuscript should ensure no ambiguity regarding this.*

**Response:** Accepted. We have added a clarifying sentence in Section 2.1 explicitly stating that this is a point-in-time systematic review based on a single search date of 22 December 2023, and that no Living Systematic Review methodology has been adopted. No scheduled search updates are planned. This removes any potential ambiguity for readers and reviewers.

**Comment 6:** *Some sections remain repetitive between Results and Discussion.*

**Response:** We have reviewed the Results and Discussion sections and removed or condensed passages where findings were restated without analytical development. The revised Discussion now advances interpretation rather than summarising results already presented in Section 3.

**Comment 7:** *Language has improved, but minor phrasing refinements could still enhance clarity.*

**Response:** We have reviewed the manuscript for phrasing and made refinements throughout, particularly in the Discussion and Conclusion sections, to improve precision and readability.

**Comment 8:** *Tables are informative but could benefit from more analytical summarization.*

**Response:** We have reviewed the tables and acknowledge this comment. Given that the primary analytical synthesis has been substantially developed in the text — particularly in Sections 4.1–4.3 and the new conceptual framework — we believe the tables now serve their intended descriptive function effectively as companions to the deepened analytical narrative. We are happy to revise specific tables further if the reviewer identifies particular areas of concern.

**Comment 9:** *The conclusion is somewhat general and could be more forward-looking.*

**Response:** Accepted. The Conclusion (Section 5) has been substantially rewritten. The revised conclusion: (1) summarises findings at the three determinant levels; (2) identifies three specific future research directions (geographic expansion beyond EU, explicit theoretical frameworks, and formal quality appraisal in future reviews); and (3) provides targeted recommendations for practitioners and policymakers regarding GI governance and consumer communication strategies.

# Reviewer report: Reviewer response for version 1

**DOI:** 10.5256/f1000research.173789.r372366

**Published:** 2025-05-02

**Authors:** Nilgun Dogan

**Affiliations:** 1 Gümüşhane University, Gümüşhane, Turkey

The academic language and English of this study seems inadequate. This situation affects over the academic value of this study as a whole. There are deficiencies in grammar and expressions in the whole text. These can be tracked in the explanations of the text signed by me. The phrasing of the sentences should be reviewed and assistance should be sought from a professional English translator (native speaker).

 The results of the study were only informative. It lacks the scientific quality of this study. The results and conclusions are repetitive. The literature review should be thorough deeply and the scientific aspect of the study should be made clear by presenting the results in a way that supports the conclusion **.**

Are the rationale for, and objectives of, the Systematic Review clearly stated?

Yes

Is the statistical analysis and its interpretation appropriate?

Partly

If this is a Living Systematic Review, is the ‘living’ method appropriate and is the search schedule clearly defined and justified? (‘Living Systematic Review’ or a variation of this term should be included in the title.)

Yes

Are sufficient details of the methods and analysis provided to allow replication by others?

Partly

Are the conclusions drawn adequately supported by the results presented in the review?

Partly

Reviewer Expertise:

My area is Agricultural economy, agricultural marketing, and I have some articles in the subject of GI-labelled agricultural products from the perspectives of the farmesr and consumers.

I confirm that I have read this submission and believe that I have an appropriate level of expertise to confirm that it is of an acceptable scientific standard, however I have significant reservations, as outlined above.

# Response

**Published:** 2026-01-10

**Authors:** AILIN TAN

**Affiliations:** Faculty of Economic and Manage, National University of Malaysia Faculty of Economic and Management, Bangi, Selangor, Malaysia

In this revised version, we substantially improved the academic language and clarity of the manuscript through comprehensive editing and rephrasing throughout the text. We also strengthened the scientific contribution by restructuring the Results, Discussion, and Conclusion sections to reduce repetition and to present findings in a clearer evidence based narrative. In addition, the literature review was reorganised to provide a deeper synthesis of prior review studies and to highlight the specific research gap addressed by this paper. Finally, we enhanced methodological transparency by reporting the PRISMA guided search, screening, and eligibility procedures in a more detailed and reproducible manner, and by clarifying how consumer responses and determinants were coded and summarised across the 64 included studies.
